# The m^6^A-methylase complex recruits TREX and regulates mRNA export

**DOI:** 10.1038/s41598-018-32310-8

**Published:** 2018-09-14

**Authors:** Simon Lesbirel, Nicolas Viphakone, Matthew Parker, Jacob Parker, Catherine Heath, Ian Sudbery, Stuart A. Wilson

**Affiliations:** Sheffield Institute For Nucleic Acids (SInFoNiA), Department of Molecular Biology and Biotechnology, University of Sheffield, Firth Court Western Bank, Sheffield, S10 2TN UK

## Abstract

*N*^6^-methyladenosine (m^6^A) is the most abundant internal modification of eukaryotic mRNA. This modification has previously been shown to alter the export kinetics for mRNAs though the molecular details surrounding this phenomenon remain poorly understood. Recruitment of the TREX mRNA export complex to mRNA is driven by transcription, 5′ capping and pre-mRNA splicing. Here we identify a fourth mechanism in human cells driving the association of TREX with mRNA involving the m^6^A methylase complex. We show that the m^6^A complex recruits TREX to m^6^A modified mRNAs and this process is essential for their efficient export. TREX also stimulates recruitment of the m^6^A reader protein YTHDC1 to the mRNA and the m^6^A complex influences the interaction of TREX with YTHDC1. Together our studies reveal a key role for TREX in the export of m^6^A modified mRNAs.

## Introduction

The transport of mRNA from the nucleus to the cytoplasm represents a key step in the eukaryotic gene expression pathway. mRNA export is closely coupled with capping, splicing and 3′ end processing of primary transcripts^1^. The integration of RNA processing and export ensure high fidelity in the gene expression process and competition between export and surveillance factors determines whether mRNAs are exported or degraded^2^. The major complex involved in mRNA export is TREX which has 14 known subunits^1^. TREX triggers the recruitment of the NXF1:NXT1 heterodimer which is required for translocation of mRNA from its site of transcription through the nuclear pore to the cytoplasm. This is achieved by adaptor (ALYREF) and co-adaptor (CHTOP and THOC5) subunits within TREX which drive NXF1 into a conformation allowing a stable interaction with mRNA^3,4^. Additional adaptor proteins which bind and recruit NXF1 to the mRNP have been identified most notably SR proteins^5,6^.

Beyond RNA processing, a number of other regulatory processes have been implicated in mRNA export control including methylation of RNA, though the molecular mechanisms are unclear. The N^6^ methyladenosine modification (m^6^A) is the most common internal methylation event in mRNA^7^. Recent work indicates that it is persistent from birth to degradation of target mRNA under steady state conditions and its addition reduces the half life of its target mRNAs^8^. m^6^A influences a number of other processes including pre-mRNA splicing, alternative polyadenylation and translation^9,10^. Genome-wide analyses using the technique of m^6^A-seq.^11,12^ and more recently cross-linking immunoprecipitation (CLIP)^13^ have revealed a distinctive landscape of m^6^A modifications on mRNAs with peaks in long internal exons and 3′ UTRs. These peaks are enriched in an RRACU consensus sequence. The heterodimeric METTL3 and METTL14 complex is responsible for the catalytic addition of the m^6^A modification upon target RNAs, often referred to as “writers”. Two further regulatory members of the m^6^A complex, WTAP and KIAA1429, are responsible for the targeting and formation of an active m^6^A complex^14,15^. RBM15 and RBM15B bind the methylase complex and recruit it to specific sites in mRNA^16^. Notably both RBM15 and RBM15B have been implicated in mRNA export control^17,18^. ZC3H13 is the most recently discovered member of the m^6^A complex which bridges the interaction between RBM15 and WTAP^19^ and maintains the nuclear localisation of the m^6^A complex^20^.

The “reader proteins” recognise and bind the m^6^A modification and include cytoplasmic YTHDF1, 2, 3^21,22^, nuclear YTHDC1^23^, and several hnRNP proteins including HNRNPC which alters splicing decisions^24^ and HNRNPA2B1 which regulates miRNA processing^25^. The cytoplasmic reader proteins play roles in translation and mRNA stability^22,26^. The nuclear reader protein, YTHDC1, has been implicated in splicing control^27^ and transcriptional silencing of XIST^16^. YTHDC1 also plays a role in the control of mRNA export where it has been proposed to work in conjunction with SRSF3 to promote export of selected transcripts^28^.

A further connection between the m^6^A machinery and mRNA export was observed following depletion of writer METTL3. This led to an alteration in the circadian clock through altered export kinetics for key mRNAs involved in this process^29^, though the molecular mechanisms involved were not described. Finally, evidence of a connection between mRNA export and m^6^A comes from a class of proteins known as “erasers” (ALKBH5 and FTO) that can remove the modification. Both are members of the same ALKB subfamily of Fe(II)/2-oxoglutarate dioxygenases. FTO preferentially recognises N^6^-2′-O-dimethyladenosine (m^6^A_m_) located adjacent to the mRNA m^7^G cap and this modification increases mRNA stability^30^. ALKBH5 is located in nuclear speckles in common with mRNA splicing and export factors and its loss is reported to lead to increased cytoplasmic poly(A)^+^ RNA signal, suggesting an alteration in mRNA export control though again the molecular mechanisms involved are unclear^31^.

In this study we reveal how the m^6^A machinery exerts control on mRNA export via TREX. The m^6^A writer complex recruits TREX to specific mRNAs and TREX stimulates recruitment of YTHDC1 to the mRNP. Knockdown of the m^6^A writer complex also stimulates TREX interactions with YTHDC1. NXF1 associates with both TREX and YTHDC1 indicating they collaborate to drive efficient export of transcripts subject to m^6^A control.

## Results

### TREX associates with the m^6^A methyltransferase complex

**Figure 1:**
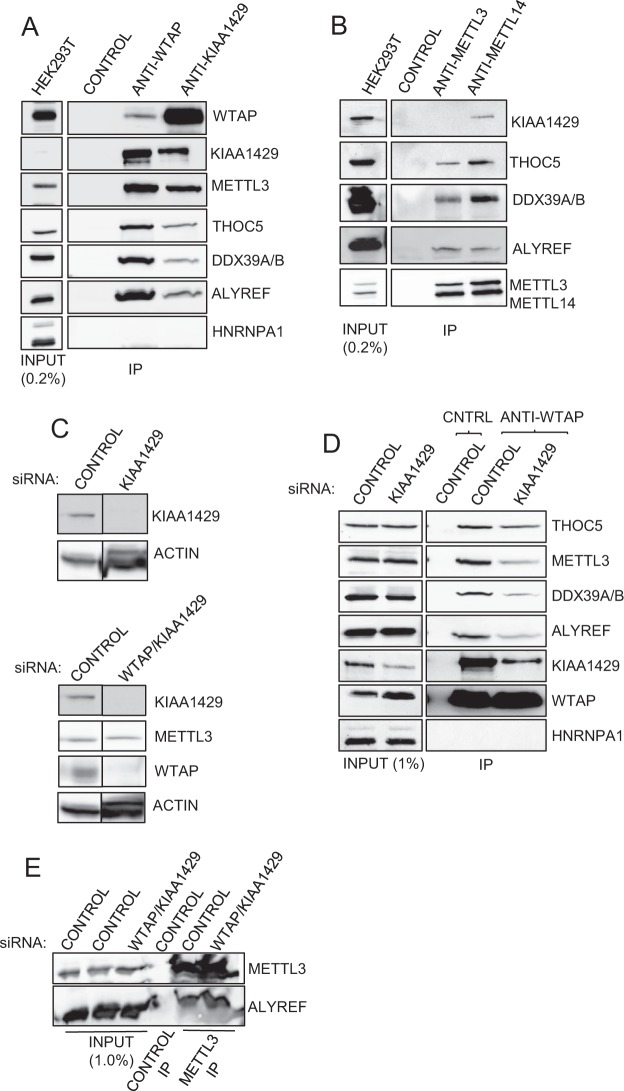
The TREX complex associates with the m^6^A methyltransferase complex. (**A**) WTAP and KIAA1429 co-IP/Western analysis with TREX complex subunits. CONTROL antibody was anti-FLAG throughout the study. Antibodies used for IP are shown above the panels and antibodies used for detection on the right hand side of panels throughout the study. (**B**) METTL3/METTL14 Co-IP/Western with TREX subunits. (**C**) Western analysis showing efficient depletion of KIAA1429 by RNAi (upper panel). Combined KIAA1429 and WTAP siRNA treatment resulted in substantial knockdown of both proteins (lower panel). (**D**) WTAP Co-IP/Western analysis in a KIAA1429 siRNA background with the indicated TREX and m^6^A machinery subunits. (**E**) METTL3 co-IP/Western analysis with ALYREF. METTL3 antibody was used for the IP. All Co-IP analysis presented in the paper were carried out in the presence of RNase A. Where separate panels are shown for same the protein in Western blots, these are all taken from the same blot at the same exposure. Full size blots are displayed in Supplementary Information.

To explore the connection between the m^6^A modification and mRNA export, we immunoprecipitated subunits of the m^6^A complex and assessed their interaction with TREX in the presence of RNase A, to disrupt interactions bridged by RNA (Fig. 1A,B). Multiple TREX subunits (ALYREF, UAP56, THOC5, CHTOP) co-immunoprecipitated (co-IP) with all four subunits of the core m^6^A complex (WTAP, METTL3, METTL14, KIAA1429). In contrast, HNRNPA1 did not, indicating that RNase A treatment prevented co-IP of general mRNP binding proteins. To further investigate these interactions we knocked down KIAA1429 using RNAi (Fig. 1C), carried out a WTAP IP and examined the impact on binding partners (Fig. 1D). Depletion of KIAA1429 significantly reduced the levels of METTL3 associated with WTAP, indicating KIAA1429 is important to maintain integrity of the core m^6^A complex. Furthermore, KIAA1429 RNAi led to reduced levels of TREX subunits associated with WTAP. However, the combined knockdown of WTAP and KIAA1429, did not disrupt the association of METTL3 with ALYREF (Fig. 1E). Therefore, the reduced levels of TREX associated with WTAP following KIAA1429 RNAi most likely reflect a reduced association of WTAP with METTL3 in that context. Whilst the levels of TREX subunits in the cell were unaffected by KIAA1429 RNAi, the levels of WTAP protein increased significantly (Fig. 1D, see inputs) as reported previously^32^, suggesting the cells attempts to compensate for KIAA1429 loss with increased WTAP levels. Together these results indicate that TREX associates with the m^6^A methylase complex.

### The m^6^A methyltransferase complex regulates nuclear export of transcripts

Given the association of the m^6^A machinery with TREX, we investigated the functional consequences of this interaction. We focussed our analysis on KIAA1429 and WTAP since subcellular fractionation showed they were associated with the chromatin and nucleoplasmic fractions, similar to TREX subunits (Fig. S1A). RNAi mediated knockdown of KIAA1429, WTAP, or KIAA1429 + WTAP gave no detectable mRNA export block using oligo (dT) fluorescence *in situ* hybridisation (FISH) (Fig. S1B). We reasoned that this may be because only a fraction of the transcriptome is subject to m^6^A control^11^. Therefore, we examined the nucleocytoplasmic distribution of selected transcripts following individual RNAi of KIAA1429 or WTAP using RT-qPCR. Transcripts were selected for analysis by cross referencing WTAP CLIP^33^, YTHDC1 CLIP^23^ and m^6^A RIPseq.^13^. By comparing all three data sets we identified methylated transcripts bound by WTAP or YTHDC1 or both (Table S1). TAF7 was chosen as a methylated intronless transcript, negating any splicing defect we may have observed. PTPN12 and DICER contain large internal m^6^A modified exons^11,13^. The transcripts GSTP1, MC1R and SYMPK were chosen as negative controls since they did not appear in our filtered list of methylated transcripts. We examined the nuclear/cytoplasmic ratio for the selected transcripts following single knockdown of either KIAA1429 or WTAP but did not see any significant differences (Fig. S2).

**Figure 2:**
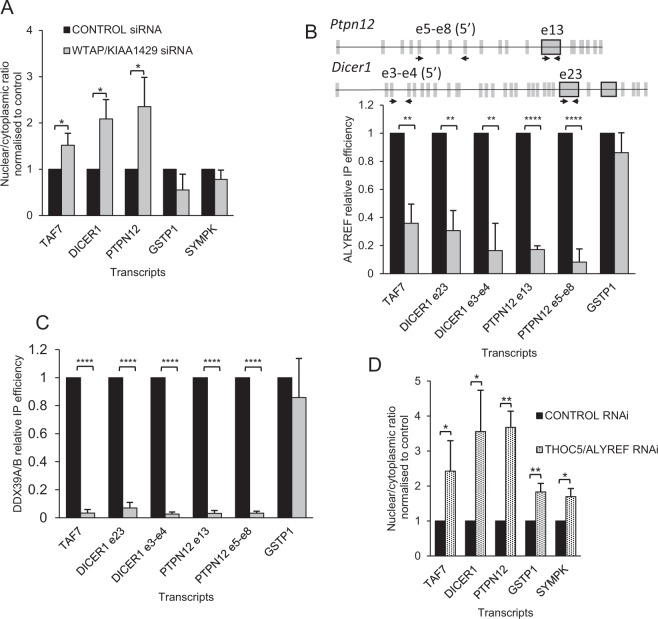
Co-knockdown of KIAA1429 and WTAP results in an export block for methylated transcripts. (**A**) qRT-qPCR analysis of m^6^A modified (TAF7 (intronless), DICER1, PTPN12 (spliced) and non-m^6^A modified mRNAs (GSTP1, SYMPK). The nuclear/cytoplasmic ratios normalised to control siRNA treated cells are shown. (**B**) ALYREF RNA immunoprecipitation (RIP) analysis by RT-qPCR are shown together with the positions of primers used on the long internal exon genes. Long internal exons with reported m^6^A sites^11^ have a black outline. (**C**) DDX39A/B RIP analysis by RT-qPCR. (**D**) RT-qPCR analysis the nuclear/cytoplasmic ratios for selected transcripts following THOC5/ALYREF combined RNAi. RT-qPCR results throughout the paper represent the averages from 3 independent experiments with standard deviations presented.

Since we had observed that KIAA1429 knockdown led to increased levels of WTAP protein, we reasoned that such a compensatory mechanism may mask any defects in mRNA export. We have observed similar compensatory mechanisms previously masking mRNA export defects for subunits of the TREX mRNA export complex^34^. Therefore, we examined the impact of a combined knockdown of WTAP and KIAA1429 on the nucleocytoplasmic distribution of selected mRNAs. siRNA treatment led to robust knockdown of both WTAP and KIAA1429 (Fig. 2A), but the reduction in the levels of these proteins did not impact on METTL3 levels. Depletion of WTAP and KIAA1429 led to a clear increase in the nuclear/cytoplasmic ratio of the spliced and intronless RNAs selected for their binding to the m^6^A complex. In contrast, transcripts with no evidence for association with this complex (GSTP1 and SYMPK), showed no significant alteration in their nuclear/cytoplasmic ratios. These data suggest that the m^6^A methylation machinery is required for efficient export of transcripts it associates with.

As TREX associates with the m^6^A methylase complex we considered that the block in mRNA export observed following WTAP/KIAA1429 RNAi might be due to inefficient loading of TREX onto mRNAs recognised by the m^6^A complex. To test this we used RNA immunoprecipitation (RIP) combined with RT-qPCR to measure the amount of mRNAs associated with the TREX subunits ALYREF and DDX39A/B (Fig. 2C,D). WTAP/KIAA1429 knockdown led to a drastic reduction in the levels of selected transcripts associated with TREX subunits ALYREF and DDX39A/B, but had no impact on the levels of the control GSTP1 mRNA bound to these proteins. For both DICER1 and PTPN12, chosen as they have long internal exons containing m^6^A sites^11^, the recruitment of ALYREF was also disrupted in regions of the mRNA not reported to have m^6^A sites (Fig. 2C). This suggests that either additional m^6^A sites are present in these mRNAs which have thus far eluded detection or alternatively that loss of ALYREF or UAP56/DDX39B at a single site on the mRNA has a propagative effect, leading to disruption of additional binding sites for these proteins on the mRNP. Double RNAi of ALYREF/THOC5 which disables TREX^34^, led to a robust increase in the nuclear-cytoplasmic ratio for transcripts known to carry the m^6^A modification (Fig. 2E). Thus establishing that mRNAs dependent on WTAP/KIAA1429 for efficient export, also require TREX. Together these data show that the m^6^A methylase complex association with certain mRNAs leads to stable binding of TREX to those mRNAs and subsequent export.

### Global analysis of WTAP/KIAA1429 knockdown on mRNA export

**Figure 3:**
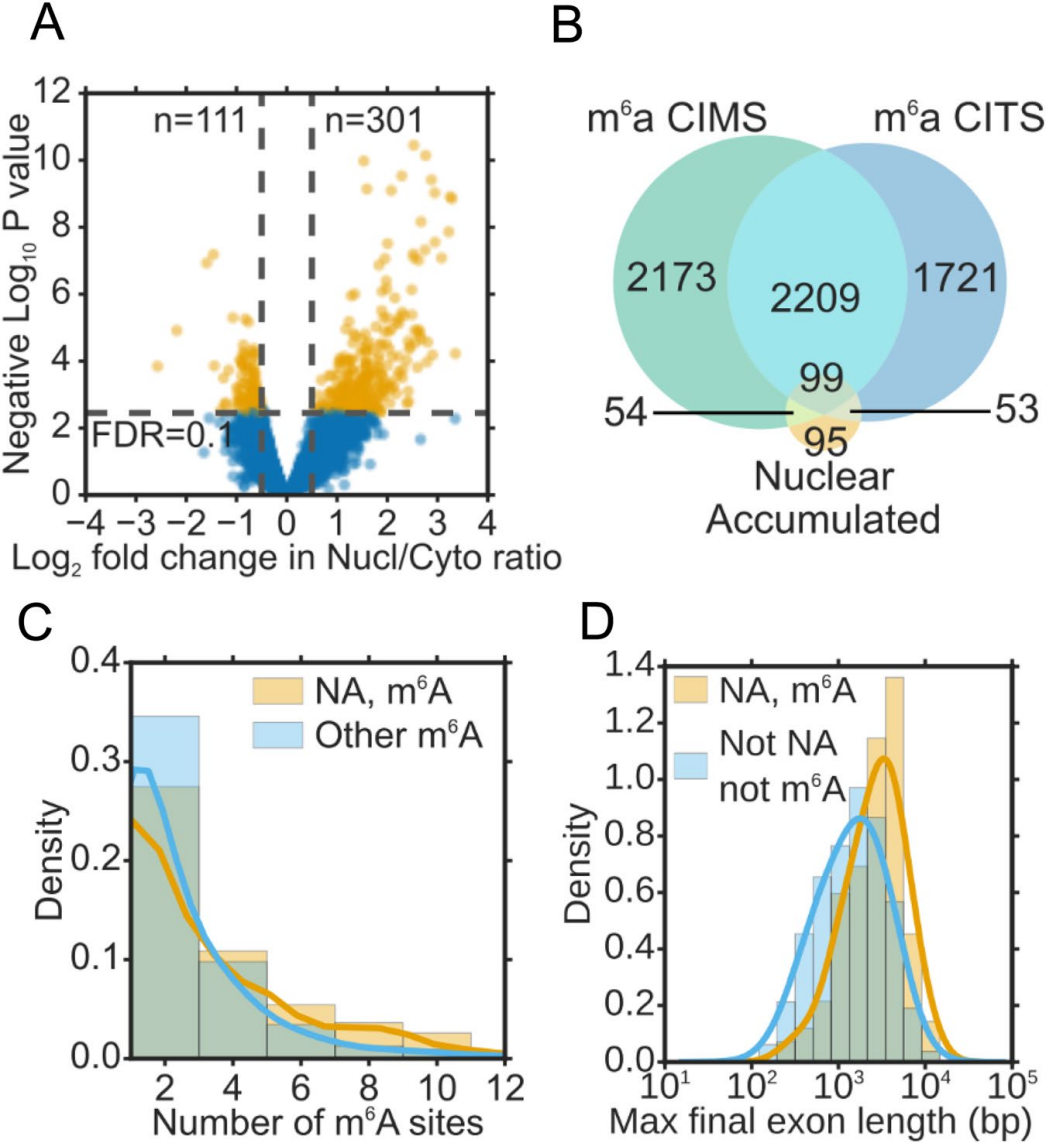
Global pattern of mRNA distribution following WTAP/KIAA1429 knockdown. (**A**) Volcano plot showing log2 fold change in nuclear/cytoplasmic ratio in WTAP/KIAA1429 knockdown vs. control RNAi, determined by RNA-seq (n = 3). A fold change of 0.5 and an FDR of 0.1 were used to identify significantly differentially localised genes. (**B**) Overlap of nuclear accumulating genes with methylated genes, as determined by m^6^A iCLIP (miCLIP)^52^. Genes were considered to be methylated if they contained one or more miCLIP peaks within an exonic region of the gene. CIMS (cross linking induced mutation site) and CITS (crosslinking-induced truncation sites) are two alternative ways of identifying m^6^A sites as described in^52^. (**C**) Number of m^6^A sites in exonic regions per gene for nuclear accumulating (NA) methylated genes, vs. other methylated genes. (**D**) Length of the longest final exon per gene (log10 scale) for NA methylated genes vs. non-NA non-methylated genes.

To extend our studies transcriptome-wide we carried out RNA-seq analysis of nuclear and cytoplasmic RNA fractions derived from cells depleted for both WTAP and KIAA1429. These studies identified 301 mRNAs whose nuclear/cytoplasmic ratio increased indicative of an mRNA export block and 111 mRNAs with a decreased nuclear/cytoplasmic ratio (Fig. 3A). Further investigation of the mRNAs with a decreased nuclear/cytoplasmic ratio revealed that 70% of them displayed a reduction in their nuclear levels and a concomitant increase in their cytoplasmic levels (Fig. S3D,E), suggesting that they are more exported. The average size of the effect on these mRNAs was much smaller than the effect on mRNAs with increased nuclear/cytoplasmic ratio (Fig. S3C,F) and these mRNAs had only a weak enrichment for genes previously reported to be methylated (1.5x enrichment, Fig. S3E). This suggests that the change to these cytoplasmically accumulating mRNAs could be an indirect effect. However, we did note an enrichment for genes annotated with the GO terms “endoplasmic reticulum” (2.9x enrichment, q-value = 0.008) and “extracellular region” (2.0x enrichment, q-value = 0.002). This is consistent with reports that mRNAs for some secreted proteins use an alternate export mechanism^35^. Focussing on the mRNAs with an increased nuclear/cytoplasmic ratio, we found a strong overlap between this group and mRNAs previously reported to have m^6^A methylation sites (206/301 genes) (Fig. 3B). Moreover, gene products which displayed an mRNA export block had higher numbers of reported methylation sites than other methylated gene products which did not show nuclear accumulation (Fig. 3C). The methylated transcripts which showed nuclear accumulation also had longer final exons on average than non-methylated transcripts which did not show an export block (Fig. 3D). Consistent with an export block, we noted that 86% of mRNAs with an increased nuclear/cytoplasmic ratio, displayed increased nuclear and decreased cytoplasmic mRNA levels (Fig. S3A,B).

**Figure 4:**
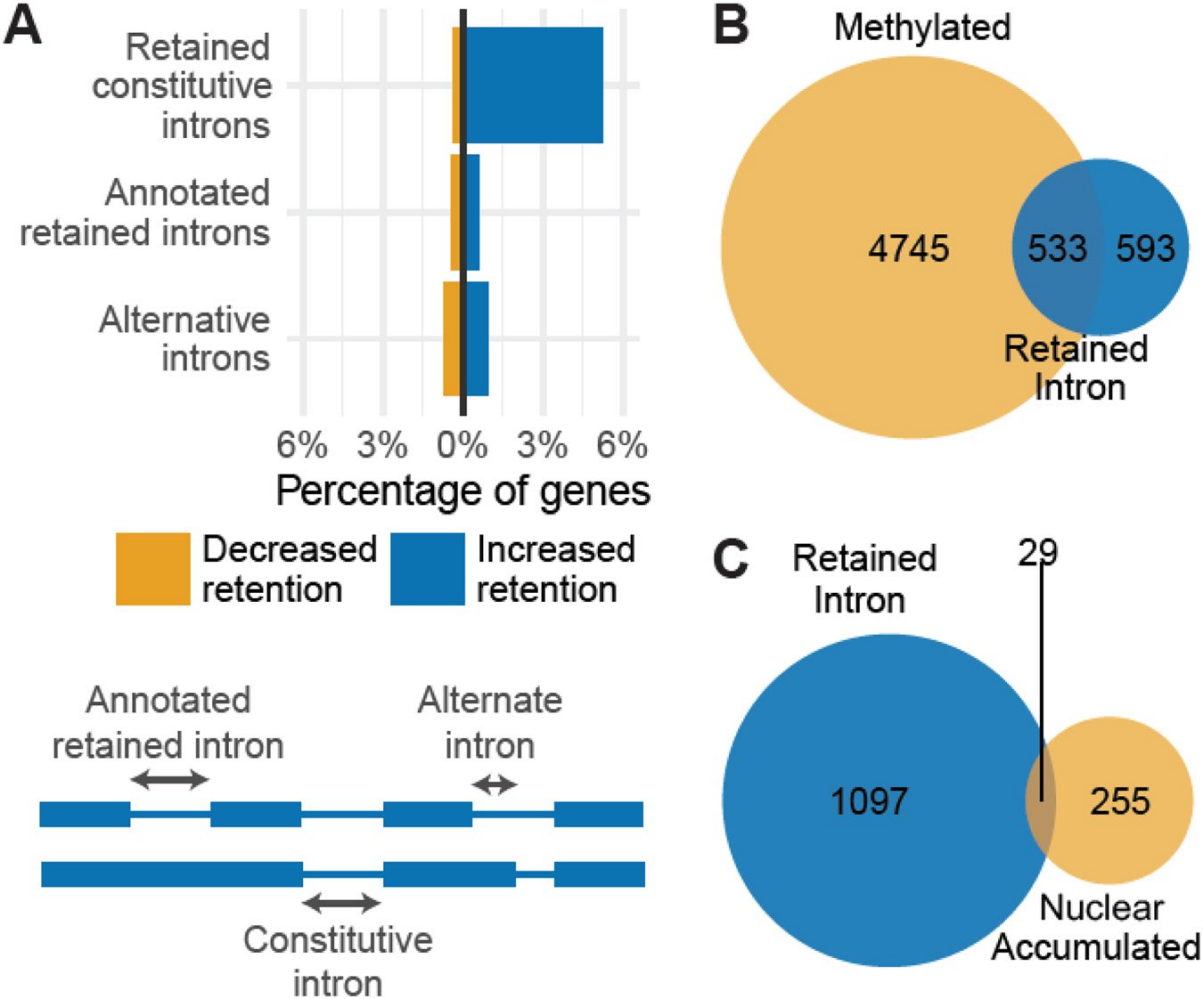
Effect of WTAP/KIAA1429 knockdown on intron retention. (**A**) Introns were divided into three categories (lower panel) – intron that is already annotated as being retained (Annotated retained intron), sequence that is always intronic in the annotation (Constitutive intron) and sequence that is sometimes intronic, sometimes exonic (Alternative intron). We measured the number of gene products containing an intron with evidence of a significant increase in intron retention (adjusted p-value < 0.1; logFC > 1) for each intron type as a fraction of gene products that contained introns of that type (upper panel). (**B**) Overlap of gene products showed to be methylated (m^6^A) with gene products showing evidence of increased intron retention on WTAP/KIAA1429 knockdown. (**C**) Overlap of gene products that show significant evidence of increased intron retention with gene products that show significant nuclear accumulation upon WTAP/KIAA1429 knockdown.

Since TREX deposition and mRNA export are coupled with splicing, we examined to what extent knockdown of WTAP/KIAA1429 altered splicing using the RNA-seq datasets. We looked at the frequency of retention of three classes of introns: constitutive, annotated retained and alternative (Fig. 4A). Retention of constitutive introns was detected in appoximately 6% of gene products, whereas annotated retained and alternative introns showed much lower increases. These data indicate that WTAP and KIAA1429 are required for correct splicing of some mRNAs as reported previously^10,36^. We examined the overlap between those gene products with retained introns and those which are reported to be methylated and found that 533 gene products with retained introns are reported to be methylated (Fig. 4B). The remaining 593 gene products with intron retention are not reported as methylated, but this may be due to poor sensitivity in detection of this modification in certain mRNAs. Alternatively, this may point to a role for these proteins in splicing not connected with their ability to trigger the m^6^A modification. We also examined the overlap between gene products which had an mRNA export block and those which had retained introns and found that only 29 gene products with an mRNA export block had altered splicing (Fig. 4C; these 29 did not include DICER1 or PTPN12). 255 genes products had no detectable splicing defect and yet had a clear mRNA export block indicating that for the majority of mRNAs, the effect on mRNA export cannot be trivially explained by a defect in splicing.

### YTHDC1 cooperates with TREX in mRNA export

**Figure 5:**
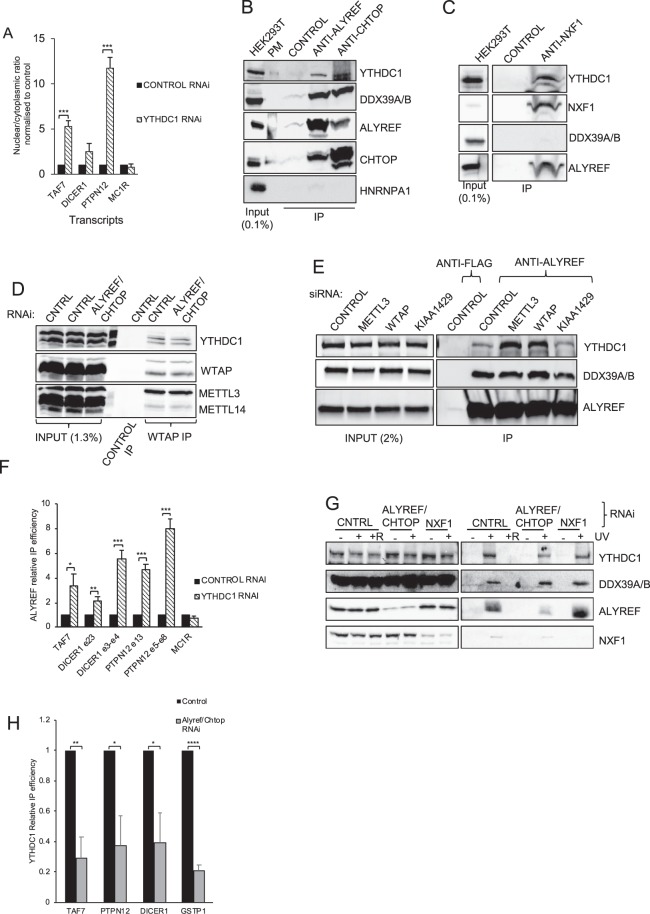
YTHDC1 associates with TREX and is required for mRNA export. (**A**) RT-qPCR analysis of the nuclear:cytoplasmic ratios for m^6^A modified (TAF7, DICER1, PTPN12) and non-modified (MC1R) transcripts following knockdown of YTHDC1. (**B**) Co-IP/Western analysis of ALYREF and CHTOP TREX subunits with YTHDC1 and other TREX subunits. PM = A lane where a protein marker was loaded. (**C**) Co-IP/Western analysis of NXF1 and YTHDC1. (**D**) WTAP co-IP/Western analysis with the indicated proteins in Control and ALYREF/CHTOP RNAi backgrounds (**E**) CO-IP/Western analysis of ALYREF with YTHDC1 and UAP56 in a background where METLL3, WTAP and KIAA1429 were knocked down using RNAi as indicated. (**F**) RT-qPCR RIP analysis of ALYREF in control RNAi and YTHDC1 RNAi cells. (**G**) UV crosslinking-mRNP capture analysis for the indicated proteins following depletion of ALYREF/CHTOP combined or NXF1. “R” indicates treatment with RNase A prior to oligo (dT) capture, as a control. (**H**) RT-qPCR RIP analysis of YTHDC1 in control and ALYREF/CHTOP RNAi cells. Where separate panels are shown for same the protein in Western blots, these are all taken from the same blot at the same exposure. Full size blots are displayed in Supplementary Information.

Recent work has shown that knockdown of the methylation reader, YTHDC1, leads to nuclear retention of YTHDC1 target transcripts^28^. We therefore investigated whether WTAP/KIAA1429 and YTHDC1 were acting in a common export pathway. We found that export targets for WTAP/KIAA1429 (Fig. 3A) are more downregulated on YTHDC1 knockdown than non-targets and the effects were similar in both cytoplasmic and nuclear RNA (Fig. S4). These data suggest that WTAP/KIAA1429 and YTHDC1 act on a common set of transcripts in mRNA export. To explore this further, we established a stable inducible YTHDC1 RNAi cell line and initially found that YTHDC1 was essential for cellular proliferation (Fig. S5B,C). We analysed the nuclear/cytoplasmic ratio following YTHDC1 RNAi and observed that TAF7, DICER1 and PTPN12, which were dependent on WTAP/KIAA1429 for mRNA export, had a dramatically increased nuclear/cytoplasmic ratio following YTHDC1 RNAi (Fig. 5A).

We noted that a recent large scale unbiased Co-IP mass spectrometry experiment analysed NXF2 as one of many baits and identified YTHDC1 as an interacting partner^37^. NXF2 has a highly restricted expression pattern and is mainly expressed in testis. Therefore, we explored whether YTHDC1 might also interact with the more prevalent and sequence related mRNA export receptor, NXF1. These experiments revealed that YTHDC1 Co-IPed with ALYREF, DDX39B and CHTOP TREX subunits together with NXF1 (Fig. 5B,C). Although YTHDC1 was present in an NXF1 IP, we could not detect NXF1 in a YTHDC1 IP using an antibody to the endogenous protein, as reported by others^28^, (data not shown), suggesting the YTHDC1 antibody might mask the region used to interact with NXF1 and TREX. Consistent with this explanation, the YTHDC1 antibody failed to immunoprecipitate any TREX subunits, in contrast to an ALYREF IP which pulled down TREX components and YTHDC1 (Fig. S5A). We conclude that TREX and NXF1 associate with YTHDC1 *in vivo*.

We further examined whether TREX might bridge the interaction between the methylase complex and YTHDC1 by immunoprecipitating WTAP in conditions where ALYREF and CHTOP were knocked down by RNAi (Fig. 5D). We found that YTHDC1 associated with WTAP and this interaction was insensitive to ALYREF/CHTOP levels. However, by immunoprecipitating ALYREF following depletion of METTL3, WTAP or KIAA1429 by RNAi (Fig. 5E), we discovered that reduced levels of either METTL3 or WTAP led to an increased association of YTHDC1 with ALYREF. These data suggest that whilst YTHDC1 and WTAP interact independently of TREX, WTAP and METTL3 regulate the association of TREX with YTHDC1. Therefore the m^6^A complex, TREX and YTHDC1 are functionally linked.

YTHDC1 recognises the m^6^A mark and may act downstream of the methylase complex. Therefore, we investigated what impact YTHDC1 depletion might have on the association of TREX with mRNA using an ALYREF RIP assay (Fig. 5F). Consistent with YTHDC1 acting downstream of ALYREF in mRNA export, we observed a significant increase in the amounts of mRNAs subject to m^6^A modification associated with ALYREF following knockdown of YTHDC1, rather than a decrease. One possible cause for increased association of ALYREF with mRNAs may be a failure to trigger the handover of mRNA to NXF1 by ALYREF which normally occurs during mRNA export^38^. Since TREX associates with the methylase complex, we also investigated whether TREX was required for efficient association of YTHDC1 with poly(A)^+^ RNAs *in vivo* (Fig. 5G). Strikingly, we found that disruption of TREX following RNAi of the two key subunits ALYREF and CHTOP, reduced the association of YTHDC1 and NXF1 with poly(A)^+^ RNAs, yet DDX39A/B association, which lies upstream of TREX in the mRNA export complex assembly pathway^1^, was unaltered. Using RIP assays we further established that knockdown of the TREX subunits ALYREF/CHTOP significantly reduced the association of YTHDC1 with selected mRNAs (Fig. 5H). Intriguingly, this effect was observed with both reported methylated transcripts (TAF7, PTPN12, DICER1) and the GSTP1 transcript which was not reported to be methylated. Further analysis of previously published YTHDC1 PAR-CLIP data^27^, revealed that of the 1123 genes reproducibly found to be YTHDC1 targets in this study, only 39% where amongst the genes reported as methylated in either of the datasets we used. This suggests either the methylation data has a very low sensitivity or that YTHDC1 is able to bind mRNAs via an additional m^6^A-independent mechanism. Since we observed no export block for GSTP1 following WTAP/KIAA1429 RNAi (Fig. 2A) this would favour the latter explanation that YTHDC1 has a broader role in mRNA metabolism which goes beyond m^6^A control. Together these data suggest a model in which the m^6^A methylase complex recruits TREX to certain mRNAs and TREX additionally stimulates the recruitment of YTHDC1 and NXF1 triggering efficient mRNA export.

## Discussion

In this study we have demonstrated an essential role for the m^6^A methylation complex in the recruitment of TREX to mRNAs modified by this complex and a further role for TREX in recruitment of YTHDC1 to mRNA. This provides a mechanistic explanation for the earlier observation that m^6^A methylation alters mRNA export kinetics^29^. TREX subunits associate with m^6^A writer proteins, therefore the consensus sequence bound by the writer complex may be as important as the m^6^A modification itself, recruiting the writer complex to specific sites, together with RBM15/15B and TREX. The m^6^A writers thus drive early events associated with the mRNP such as TREX recruitment. TREX is further required for the efficient association of the m^6^A reader YTHDC1 with mRNAs and therefore TREX plays multiple roles in the mRNP interactions and exchanges which occur on mRNAs.

Why mRNAs which are subject to the m^6^A modification are dependent on the presence of the m^6^A methylase complex for recruitment of TREX whilst seemingly similar mRNAs without the m^6^A mark recruit TREX and get exported efficiently, is not clear. It may be the case that this modification allows recruitment of TREX to sites on mRNA which would otherwise be devoid of TREX. ALYREF is found along the entire body of mRNAs^39^ and it associates with the exon junction complex (EJC) protein eIF4A3 which has a preferential binding site ~24 bases upstream from exon-exon junctions^40,41^. Therefore, long internal exons and 3′ UTRs which are enriched in m^6^A sites, may naturally be devoid of TREX recruitment options due to reduced levels of EJCs in the vicinity. Therefore, the m^6^A writer complex may act as a surrogate recruitment mechanism for TREX in these regions and facilitate subsequent recruitment of NXF1 to ensure efficient translocation of the mRNP through the nuclear pore. Consistent with this notion, we found that the m^6^A modified transcripts which showed nuclear accumulation following loss of the m^6^A writer complex had longer final exons on average (Fig. 3D).

An additional intriguing observation is that depletion of the methylase complex, triggers loss of TREX at multiple sites along an mRNA (Fig. 2D). This may be because m^6^A sites are under reported with existing technologies. Alternatively, TREX deposition at a single site may encourage stable binding of TREX at other sites along the mRNP, or it could be a combination of these effects. The EJC has been reported to form self-interactions and is proposed to aid with packaging of the mRNP^42^. Similarly, ALYREF is reported to interact with itself^43^ and may help drive assembly of a compact mRNP with TREX-mRNP interactions stabilised by TREX-TREX interactions.

YTHDC1 was recently proposed to work in conjunction with SRSF3 in mRNA export and function in a complex with NXF1^28^. We now show that TREX is an essential component for export of m^6^A modified mRNAs. When TREX associates with NXF1 it induces a conformational change such that it increases the RNA binding affinity up to ten fold^3^. However, this is a two component effect with ALYREF and SR proteins in isolation only able to increase the RNA binding activity of NXF1 approximately four fold^38^. The additional RNA binding affinity associated with TREX recruitment comes from co-adaptor proteins within TREX such as THOC5 and CHTOP which associate with the NTF2L domain of NXF1. This disrupts the sequestration of the N-terminal NXF1 RNA binding domain^3,4^ leading to maximal RNA binding activity and stable association of NXF1 with the mRNP. Therefore, it is highly likely that SR proteins work in conjunction with co-adaptor proteins or even a complete TREX complex to facilitate NXF1 recruitment to m^6^A modified and non-modified mRNAs. Consistent with this, yeast SR proteins are known to associate with TREX^44^. In summary, we have identified an essential function for the m^6^A methylation machinery in mRNA export and demonstrated that this function is mediated through TREX.

## Methods

### siRNA transfections

siRNA transfections of 60% confluent 293 T cells grown in Dulbecco’s Modified Eagles Medium were carried out using RNAiMAX (Life technologies) with siRNAs at 10 nM. Silencer select siRNA ID S24832 Cat No. 4392420 targeting KIAA1429 was purchased from ThermoFisher Scientific. The siRNA targeting WTAP was described previously^16^ and had the following sequence: 5′-GGCAAGAGAUGAGUUAAUUCUAAGA. For METTL3, Stealth siRNA HS125548 from Thermofischer Scientific was used. The control siRNA was described previously^45^. siRNA transfected cells were incubated for 72 hours undergoing a second transfection at 48 hours. YTHDC1 RNAi was carried out using a stably integrated miR30 mimic RNAi cassette in Flp-In HEK293 cells as described previously^34^. The cassette contained two hairpins targeting the following sequences 5′ GAGAATGTGTCTCTTGCCAAA and 5′ CAGAGATAAACGAGTACATGA. The NXF1, ALYREF/CHTOP and Control inducible RNAi cell lines have been described previously^3,4^. Growth of YTHDC1 RNAi cells was assessed using an MTT assay^46^.

### Fluorescence In-Site Hybridisation (FISH)

FISH was carried out as described previously^38^.

### Co-IPs

Each sample used 100 µl Protein-G DYNA beads blocked over night at 4 °C in 500 µl of IP lysis buffer (50 mM HEPES-NaOH pH 7.5, 100 mM NaCl, 1 mM EDTA pH 8.0, 0.1% Triton X-100, 10% Glycerol, 1 mM DTT) containing 1% BSA and 2–10 µg of antibody. Beads were subsequently washed 3 times in 1 ml IP lysis buffer. Cells were washed with PBS before lysis in IP lysis buffer containing protease inhibitor, DNase 1, RNase A and DTT. Extracts were cleared by centrifugation at 13 200 rpm, 5 minutes at 4 °C. Cell extracts were incubated with antibody bound beads for 2 hours at 4 °C. Beads were washed 3 times in 1 ml ice cold IP lysis buffer. After the final wash beads were resuspend in 72 µl 1 M Arginine-HCl pH 3.5, incubated for 1 minute on ice and spun for 1 minute at 400 × g. Eluate was transferred to a fresh 1.5 ml tube and neutralised with 3 µl 1.5 M Tris.HCl (pH 8.8) prior to SDS-PAGE. Antibodies used in this study were: FLAG – Sigma F3165, WTAP Santa Cruz sc-374280 (used for Fig. 1A), WTAP Abcam Ab195380 used for remaining Figures, KIAA1429 – Proteintech 25712-1-AP, METTL3 – Proteintech 15073-1-AP, HNRNPA1 – Millipore 04-1469, METTL14 – Sigma HPA038002, ACTIN – Sigma A5060, YTHDC1 – Proteintech 14392-1-AP, SSRP1 – Biolegend 609701, TUBULIN – Sigma T5168, ALYREF – Abcam Ab6141, NXF1 – Abcam Ab50609, DDX39A/B and THOC5 were described previously^3^.

### RNA/Protein extraction from 293 T cells

2 × 10 cm dishes were used and nuclear/cytoplasmic fractionations were performed as described previously^47^. Nuclei obtained with this procedure were fractionated into nucleoplasm and chromatin as described previously^48^. The RNA content from each fraction was extracted using TRIzol. RNA samples were DNase-treated (TurboDNase, Ambion). Fractionations were assessed by western blot analysis, using histone H3, as a chromatin marker, SSRP1 as nuclear marker, and TUBULIN as a cytoplasmic marker.

### Formaldehyde RNA Immuno-Precipitation (faRIP)

One 6-cm dish (or 2 × 6-cm dishes for siRNA treatments) was seeded per RIP condition with 3 × 10^5^ cells/dish. Protein-RNA complexes were crosslinked *in vivo* by incubating cells with 3 mL of PBS-Formaldehyde (0.1%), as described previously^49^. 100 µL of protein G-Dynabeads^®^ beads were prepared by initial washing with 3 × 1 mL RIP lysis buffer (50 mM HEPES-HCl pH 7.5, 150 mM NaCl, 10% glycerol 1% NP-40, 0.1% SDS, and 0.5% sodium deoxycholate) before being blocked and loaded with the relevant antibody (2–10 µg diluted in 0.3 mL of RIP lysis buffer +1% BSA w/v final) for 1 hour at room temperature. Beads were washed with 3 × 1 mL RIP lysis buffer and left on ice. Each cell pellet was lysed in 400 µL RIP lysis buffer supplemented with 1 mM DTT, protease inhibitors (SigmaFAST, Sigma), 2 µL of RNase inhibitors (Ribosafe, Bioline) and 2 µL/mL of Turbo DNase (Ambion). Samples were sonicated using a Bioruptor (High, 5 × [30s-ON/30s-OFF]) and cleared by centrifugation (16100 x g, 10 min, 4 °C). 300 µL of each sample was incubated with Dynabeads for 2 hours at 4 °C and 30 µL of lysate was kept as input (10%). Following incubation, the beads were washed with 2 × 1 mL RIP lysis buffer, 2 × 1 mL high salt RIP lysis buffer (adjusted to 500 mM NaCl, 5 min each on ice), and 2 × 1 mL RIP lysis buffer. Elutions were performed as described previously^49^. The RNA content of the resulting eluates and inputs were extracted using TRIzol. RNA samples were DNase-treated (Turbo DNase, Ambion) and used for cDNA synthesis and RT-qPCR analysis. Oligonucleotides used for RT-qPCR analysis were as follows: TAF7 Forward 5′ GCCTCTACTGTGAGAAGGGG, TAF7 Reverse 5′ CACGGTCCACTCTGACGATT, DICER1 e23 Forward 5′ CATAGCGGACTGTGTGTGGAAG, DICER1 e23 Reverse 5′ AGCAGCACAGCTCACTGAAA, DICER1 e3 Forward 5′ GACTTGCTATGTCGCCTTGA, DICER1 e4 Reverse 5′ ATGCGAGGACATGATGGA, PTPN12 e13 Forward 5′ ACTCTGATGGTGCTGTGACC, PTPN12 e13 Reverse 5′ GCACCTGAATGTGTTGTTCC, PTPN12 e5 Forward 5′ GAGCGCTATTGGCCTTTG, PTPN12 e8 Reverse 5′ AATGGCACCTGTTCTTCCAC, GSTP1 Forward 5′ CCGTGGTCTATTTCCCAGTT, GSTP1 Reverse 5′ AGGTGACGCAGGATGGTATT, SYMPK Forward 5′ AACCAGACCAGAAGGATGG, SYMPK Reverse 5′ AGTACTTGCGGACCACCTC, MC1R Forward 5′ GGACCGCTACATCTCCATCT, MC1R Reverse 5′ GCATAGCCAGGAAGAAGACC, U1 Forward 5′ ACCTGGCAGGGGAGATACC, U1 Reverse 5′ GGGGAAAGCGCGAACGCAGT. IP efficiencies were calculated relative to the input RNA levels.

### mRNP capture assays

1 × 15 cm dish of cells was used per condition. 2 × binding buffer (BB) (20 mM HEPES-K-NaOH pH 7.5, 1 M NaCl, 1% SDS, 0.2 mM EDTA pH 8) was preheated to 37 °C. 1 × binding buffer was made by mixing 1:1 mRNP lysis buffer (LB) (50 mM HEPES-K-NaOH pH 7.5, 100 mM NaCl, 1 mM DTT, 1 mM EDTA pH 8.0, 0.5% Igepal Ca-630/NP-40, 0.5% Na-deoxycholate, 10% glycerol) with preheated 2 x BB. 100 µl of magnetic oligo dT beads (New England Biolabs) were used per sample and washed 3 times in 1 × BB. Cells were crosslinked with 300 mJ/cm^2^ ultraviolet light (UV). Each dish of cells was lysed in 600 µl LB supplemented with protease inhibitor cocktail and RNase inhibitors. Post lysis, samples were spun at 4 °C, 16100 g for 10 minutes. Samples were denatured by adding 1:1 lysate and 2 × BB at 37 °C. Denatured lysates were added to the beads and incubated for 1 hour at 25 °C. Samples were washed 3 times in 1 x BB and eluted in 60 µl of mRNP elution buffer (10 mM Tris pH 7.5, 1 mM EDTA pH 8) supplemented with RNase A (50 µg/mL^−1^). Elution was carried out at 25 °C shaking at 800 rpm for 30 minutes.

### mRNA Sequencing

Libraries for sequencing were prepared using the Lexogen SENSE mRNA-Seq Library Prep according to the manufacturer’s instructions and sequenced using a Hiseq2500.

### Computational Analysis

Read Preprocessing. Nine nucleotides were removed from read 1 fastq files, and six were removed from read 2, as suggested by the Lexogen SENSE library preparation manual using the cutadapt tool^50^. Samples split across two lanes were merged into a single fastq file. Transcript Abundance Quantification. Transcript abundances were estimated using salmon^51^ against the Ensembl v75 annotation (as the hg19 genome was used for the m6A iCLIP data)^52^. Abundances were then aggregated to gene level using tximport^53^. Differential Nuclear Retention Analysis. Differential nuclear retention of genes was calculated using edgeR^54^, using the contrast formula: (KD_nuc_/KD_cyt_)/(WT_nuc_/WT_cyt_). Genes with a log fold change greater than 0.5 and an FDR less than 0.1 were considered significantly accumulating in the nucleus, and used for downstream analyses. Overlap with m^6^A iCLIP data: RNA methylation sites were obtained as bed files from an m^6^A iCLIP (miCLIP) dataset^52^. The number of miCLIP sites overlapping exonic regions of each gene was counted using bedtools intersect^55^. Any gene that contained at least one overlapping miCLIP site from either m6A antibody was considered to be methylated. P-values for the intersection of methylated genes with nuclear accumulating genes were calculated using hypergeometric tests. Intron Retention Analysis: Reads were mapped to hg19 using HiSat^56^ with default parameters. Gene models were obtained from Ensembl v75 and divided into minimally spanning transcript chunks using the gtf2gtf program from the CGAT suite^57^ and annotated as either constitutive exon, constitutive intron, annotated retained intron or alternate (see code). Read counts were calculated using featureCounts from the subread package^58^ and differential chunk usage calculated using DEXSeq.^59^. Intron chunks with and adjust p-value less than 0.1 and a log_2_ fold change between control and WTAP/KIAA1429 > 1 were designated as significantly more retained. Further Analysis: Final exon length distributions were calculated using custom python scripts which are available from Zenodo, 10.5281/zenodo.1194638.

## Electronic supplementary material

supplementary data

supplementary table 1

## Data Availability

The RNA-seq data have been submitted to GEO Accession Number: GSE111878.
