# Supplementary material for: The m6A-methylase complex recruits TREX and regulates mRNA export
Source: Sci Rep. 2018 Sep 14;8:13827. doi: 10.1038/s41598-018-32310-8 (PMC6138711; doi:10.1038/s41598-018-32310-8)
Supplement: Supplementary file 1 — supplementary data [file 41598_2018_32310_MOESM1_ESM.pdf]

Title: The m^{6}A-methylase complex recruits TREX and regulates mRNA export.

Authors: Simon Lesbirel[a], Nicolas Viphakone[a], Matthew Parker[a], Jacob Parker[a], Catherine Heath[a], Ian Sudbery[a] and Stuart A. Wilson[a,*]

[a]Sheffield Institute For Nucleic Acids (SInFoNiA), Department of Molecular Biology and Biotechnology, University of Sheffield, Firth Court Western Bank, Sheffield, S10 2TN, U.K.

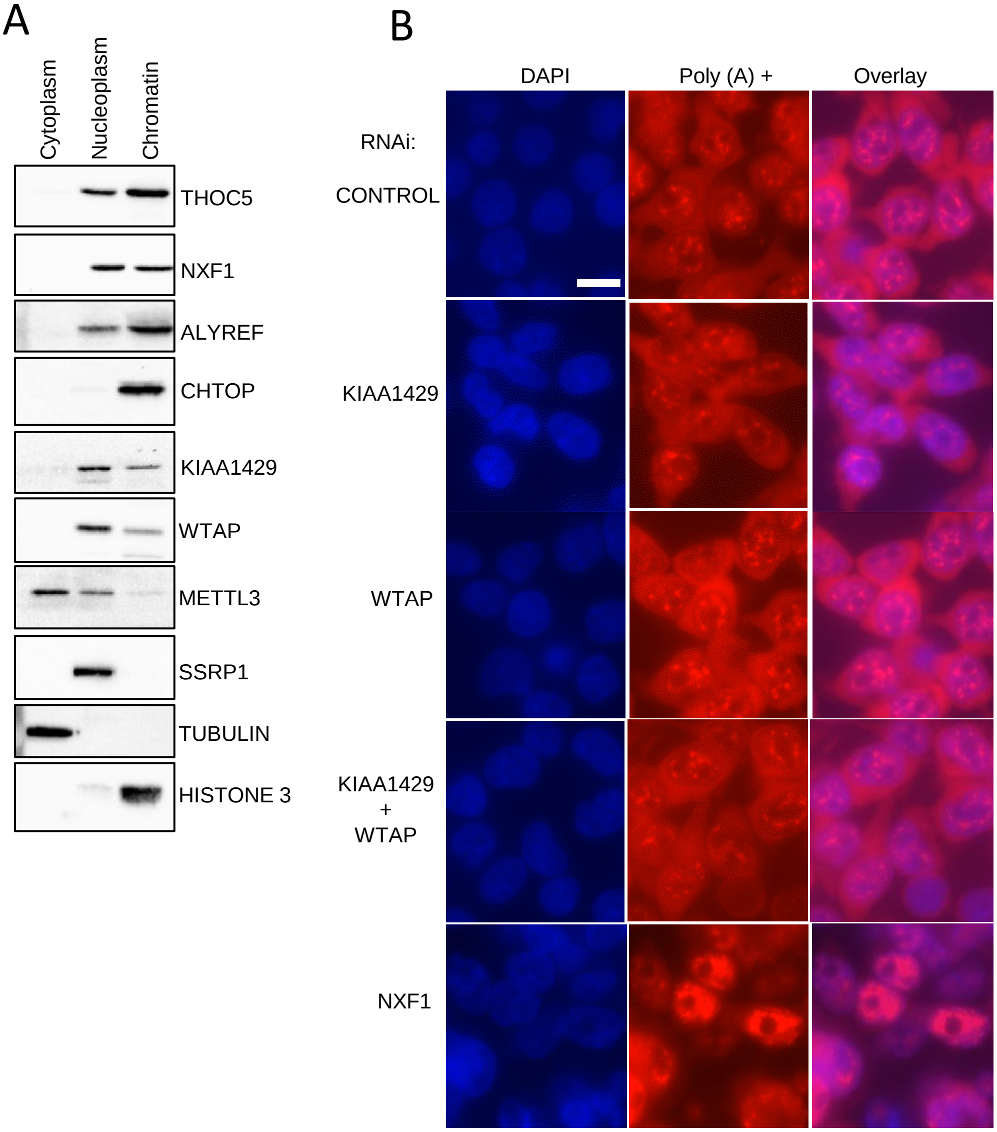

Fig. S1. Analysis of KIAA1429 and WTAP in mRNA export. A) Western analysis of subcellular fractions for the indicated proteins. SSRP1, Histone H3 and Tubulin were used as markers for each of the three subcellular fractions. B) oligodT fluorescence *in situ* hybridisation analysis of cells following knockdown of the indicated genes by RNAi. For the NXF1 RNAi a stable cell line was used which expressed a miRNA targeting NXF1 as described previously[3] . The scale bar is 10µM.

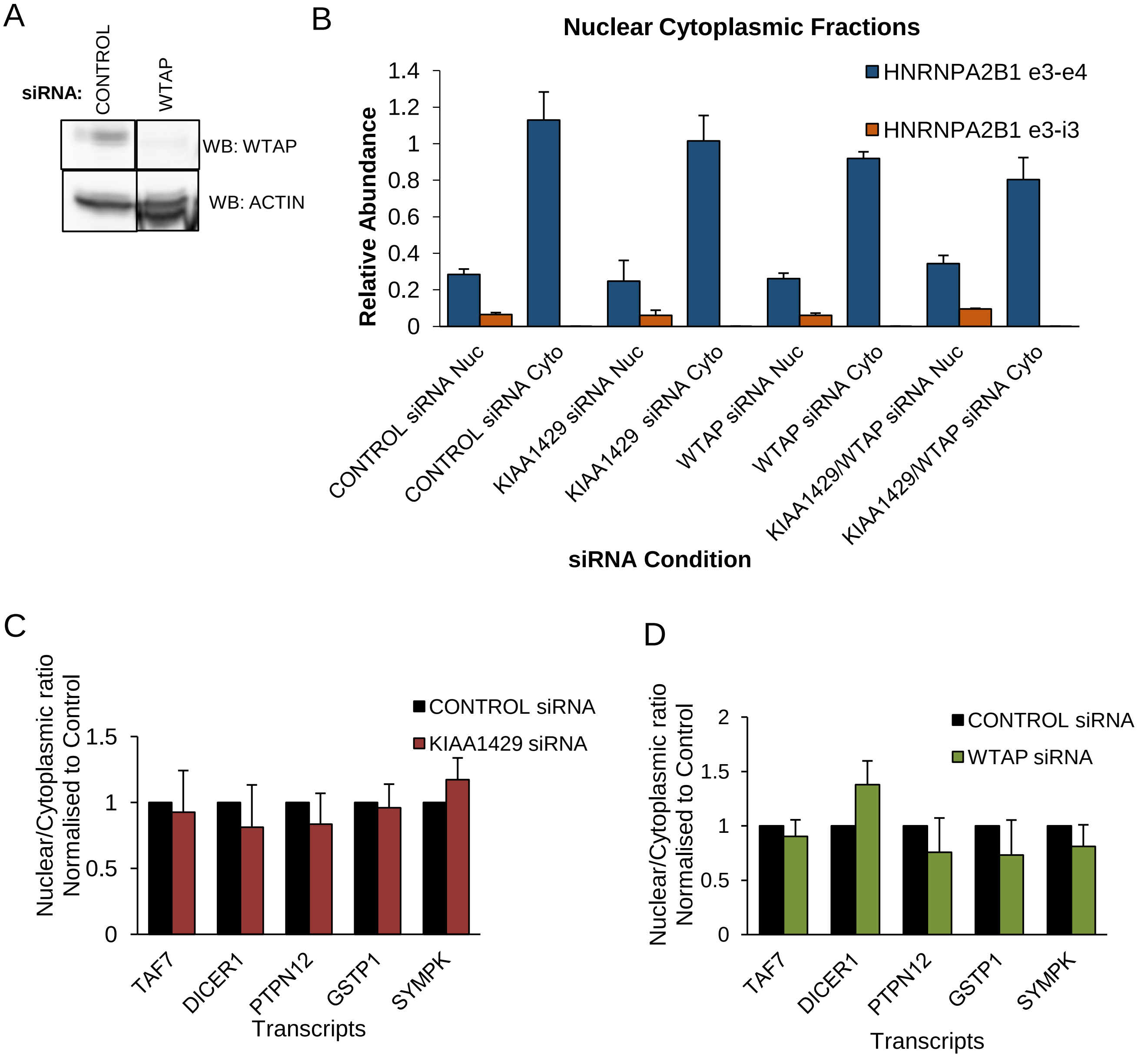

Fig. S2. mRNA Analysis of KIAA1429 and WTAP in mRNA export. A) Western analysis showing efficient knockdown of WTAP following transfection of cells with WTAP siRNA. B) qRT-PCR analysis showing efficient fractionation of subcellular compartments. The unspliced pre-mRNA is only detected in the nuclear fractions whereas the spliced mRNA is detected in both subcellular fractions. C,D) qRT-PCR analysis of nuclear/cytoplasmic ratios for selected transcripts (m^6A modified: TAF7, PTPN12, DICER1; non-modified: GSTP1, SYMPK) following individual knockdown of either WTAP or KIAA1429. Where separate panels are shown for the same protein in Fig. S1A, the panels were taken from the same blot at the same exposure.

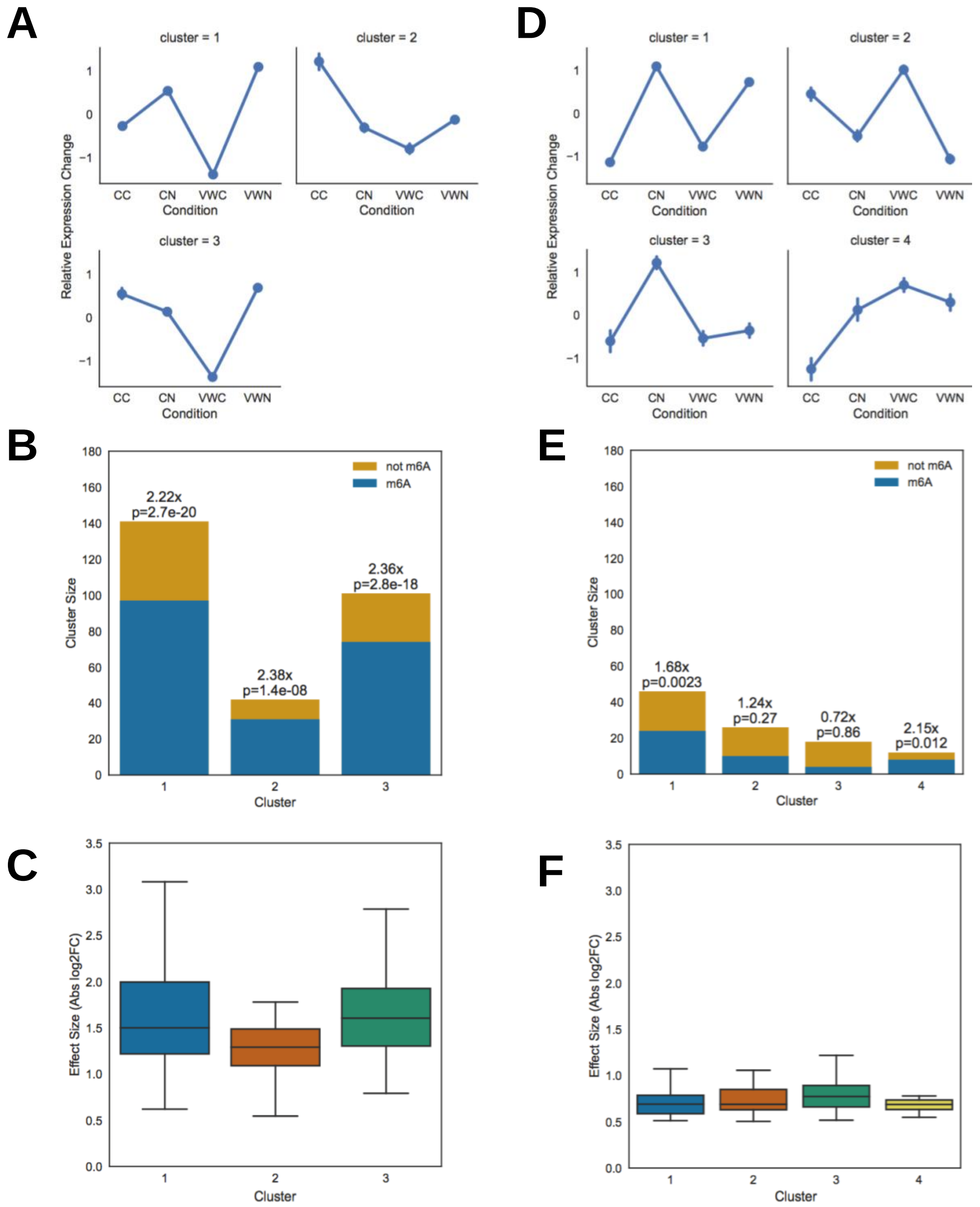

Fig. S3: Patterns of gene expression in differentially retained or exported genes. A) K-means clustering of nuclear accumulating genes suggests three clusters, showing two main patterns of gene expression. Clusters one and three contain genes with nuclear/cytoplasmic ratios greater than one and less than one, respectively, which show nuclear accumulation (increase in nuclear level and reduction in cytoplasmic level) during WTAP/KIAA1429 knockdown. Cluster two contains genes which exhibit reduction in cytoplasmic level with little or no increase in nuclear level. B) Cluster sizes for nuclear accumulating gene clusters. 86% of genes show a pattern of nuclear accumulation and cytoplasmic reduction, suggesting the majority of the effect is caused by an mRNA export block. All clusters show similarly high levels of methylation detection in the miCLIP dataset. Fold enrichment of methylation in clusters over all genes and hypogeometric p-value for enrichment are shown above the bars C) Distribution of changes in nuclear accumulation for each cluster. D, E and F) As A, B and C but for cytoplasmically accumulating genes. Clusters one and two: genes with nuclear/cytoplasmic ratios of greater than one and less than one, respectively and reduced nuclear levels and increased cytoplasmic levels following WTAP/KIAA1429 RNAi. Cluster three: genes with reduced nuclear expression and no significant change in cytoplasmic levels. Cluster four: genes which show an increase in cytoplasmic expression with no corresponding reduction in nuclear expression. 70% of genes show a pattern of cytoplasmic accumulation and nuclear reduction, suggesting the majority of the effect is caused by an increase in export. Throughout the Figure: CC = Control cytoplasmic fraction, CN = Control Nuclear Fraction, VWC = KIAA1429/WTAP RNAi cytoplasmic fraction, VWN = KIAA1429/WTAP RNAi nuclear fraction.

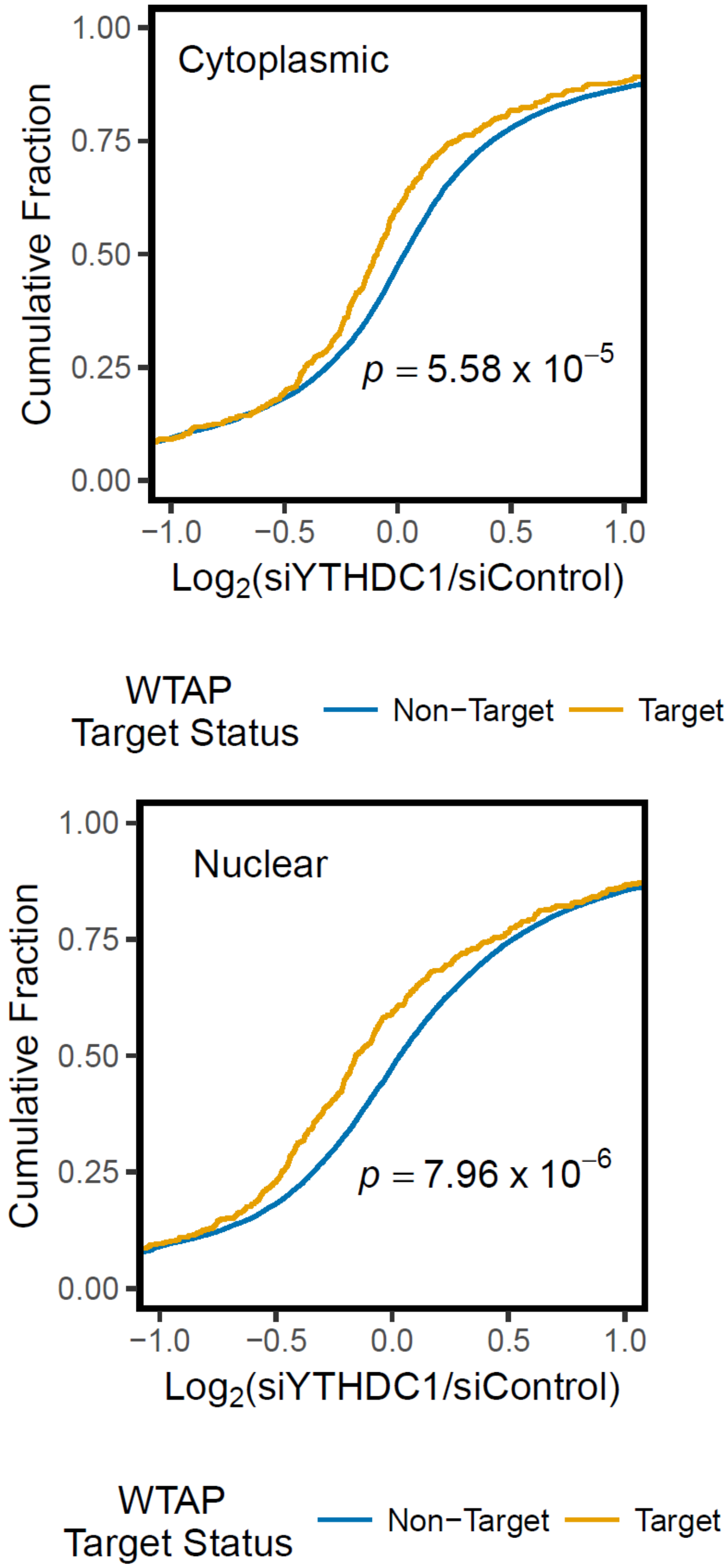

Fig. S4. WTAP/KIAA1429 targets are down-regulated on YTHDC1 knockdown. Plots show the cumulative distribution function of log-fold changes in transcript expression on YTHDC1 knockdown using data from (eLife 2017;6:e31311 DOI: 10.7554/ELIFE.31311). Log-fold change calculated as the average ratio of expression between knockdown and control for two different YTHDC1 siRNAs for both nuclear and cytoplasmic RNA fractions as indicated. Functions shown separately for nuclear retention targets of WTAP/KIAA1429 and non-targets. P-value for the difference between targets and non-targets calculated using the Mann-Whitney U test.

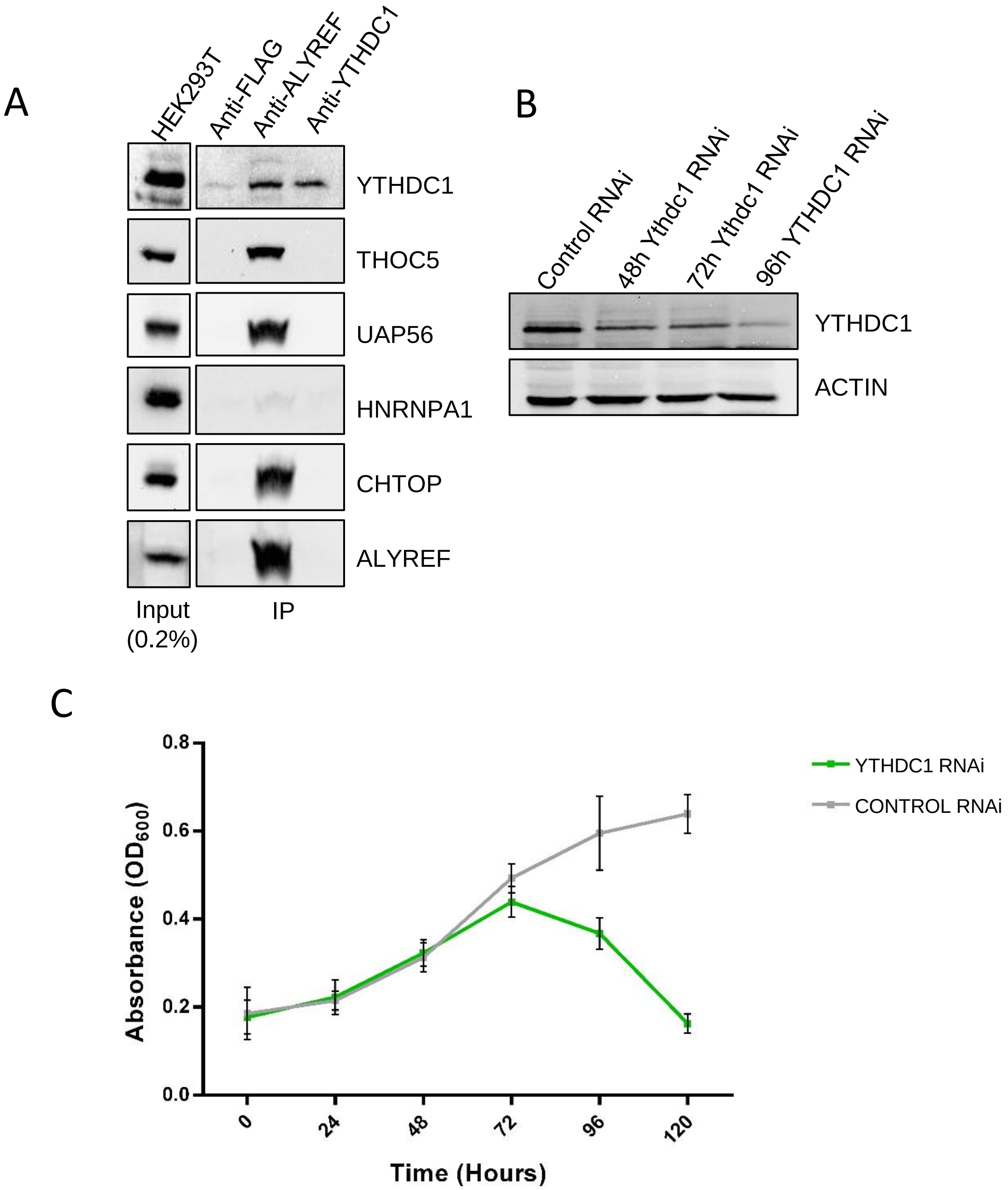

Figure S5. **Analysis of YTHDC1.** A) Co-IP western analysis for ALYREF and YTHDC1 with the indicated antibodies. B) Western blot analysis of the control and YTHDC1 stable inducible RNAi cell line. The length of time of tetracycline induction of the control or YTHDC1 hairpin is indicated. C) MTT cell proliferation assay was used to follow growth of the stable inducible RNAi cell line following tetracycline induction of the RNAi hairpin for the indicated times. The results represent the average of 3 independent experiments and the standard deviation of the mean is presented. Where separate panels are shown for the same protein in Fig. S4A, the panels were taken from the same blot at the same exposure.

Original images of blots displayed in Fig 1A.
Regions used for Figures are boxed in red

WTAP

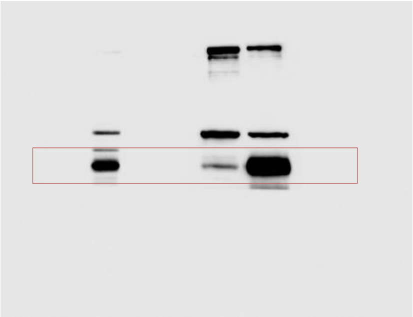

KIAA1429

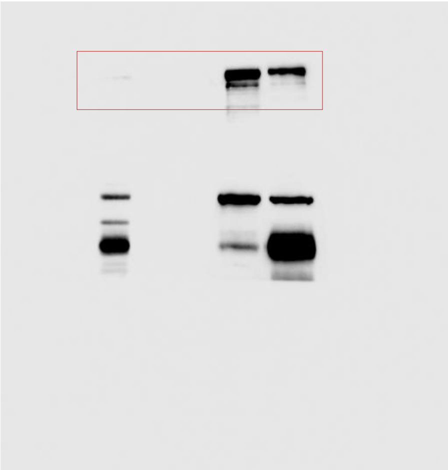

METTL3

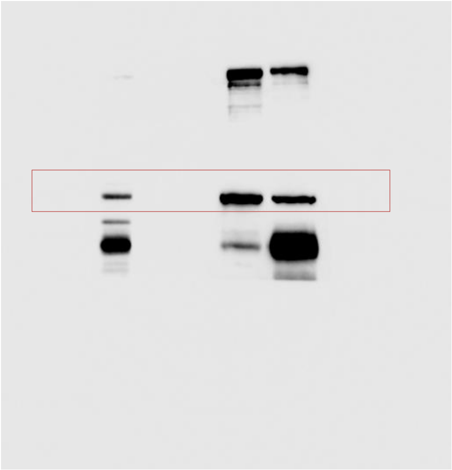

THOC5

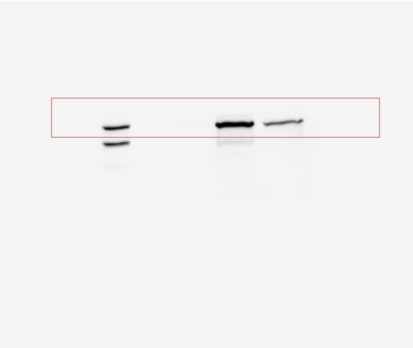

DDX39A/B

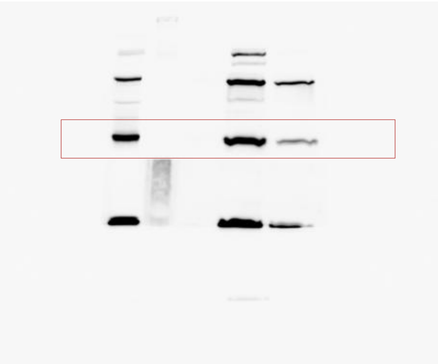

ALYREF

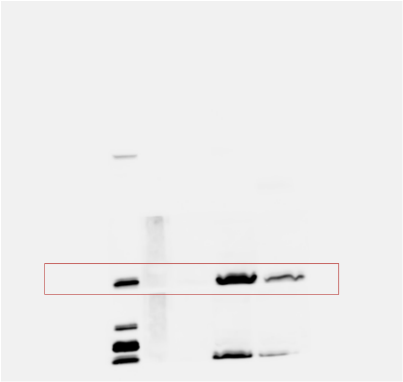

HNRNPA1

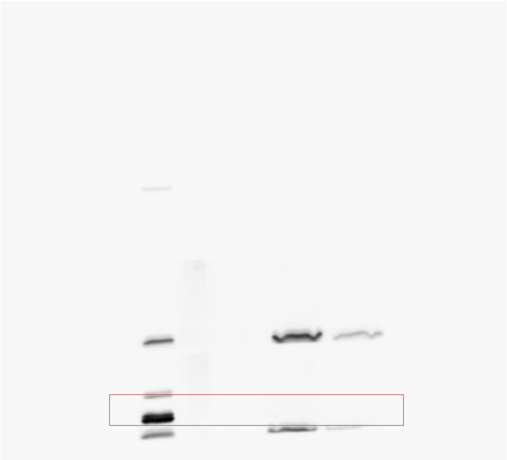

Original images of blots displayed in Fig 1B.

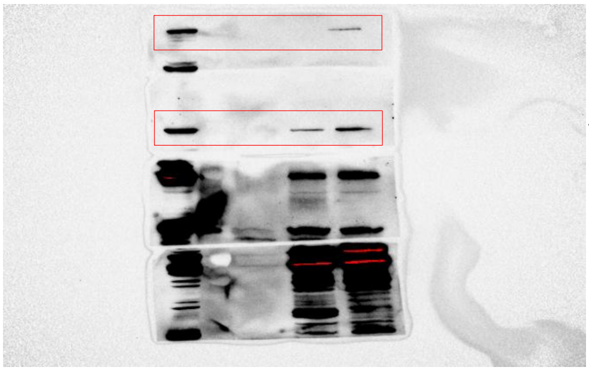

KIAA1429

THOC5

DDX39A/B

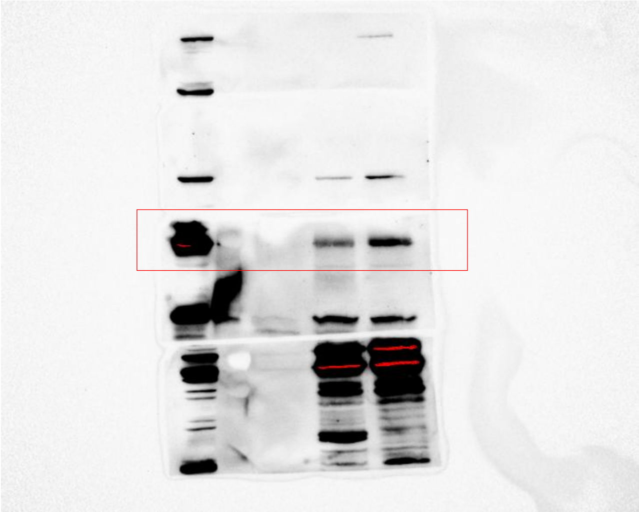

METTL3/14

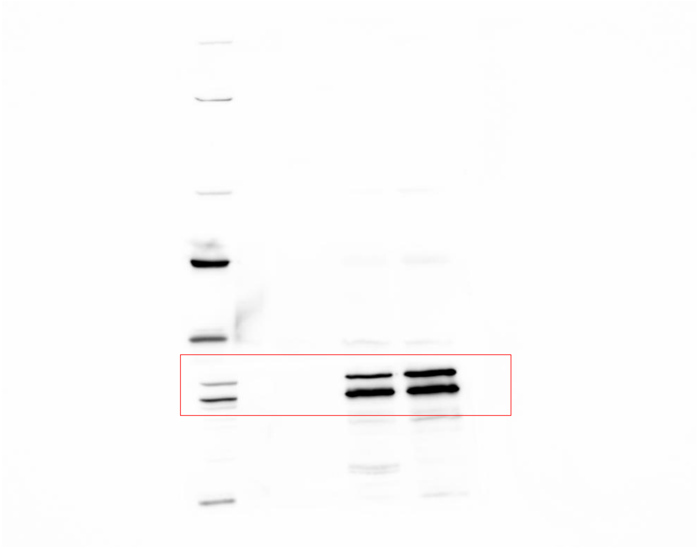

ALYREF

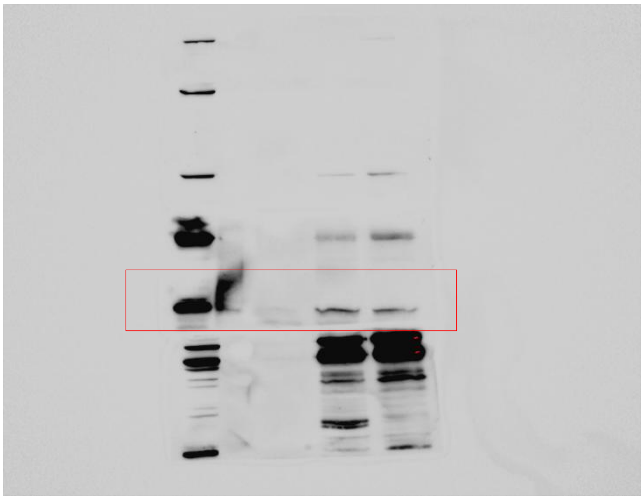

Original images of blots displayed in Fig 1C (upper panel) and S2A.

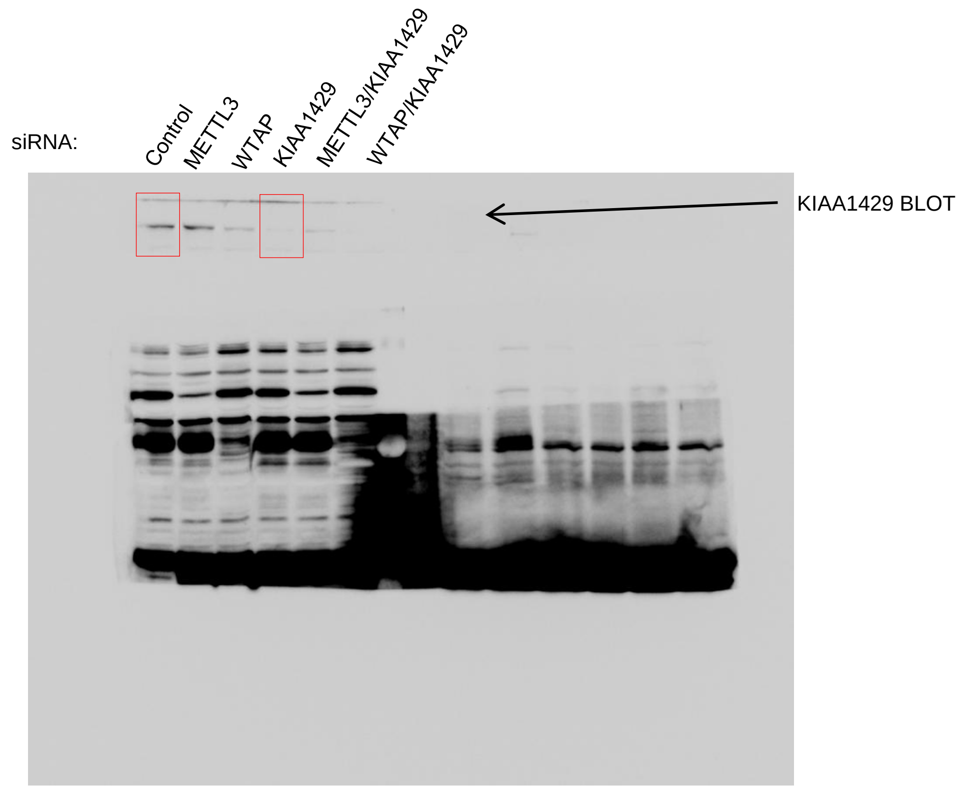

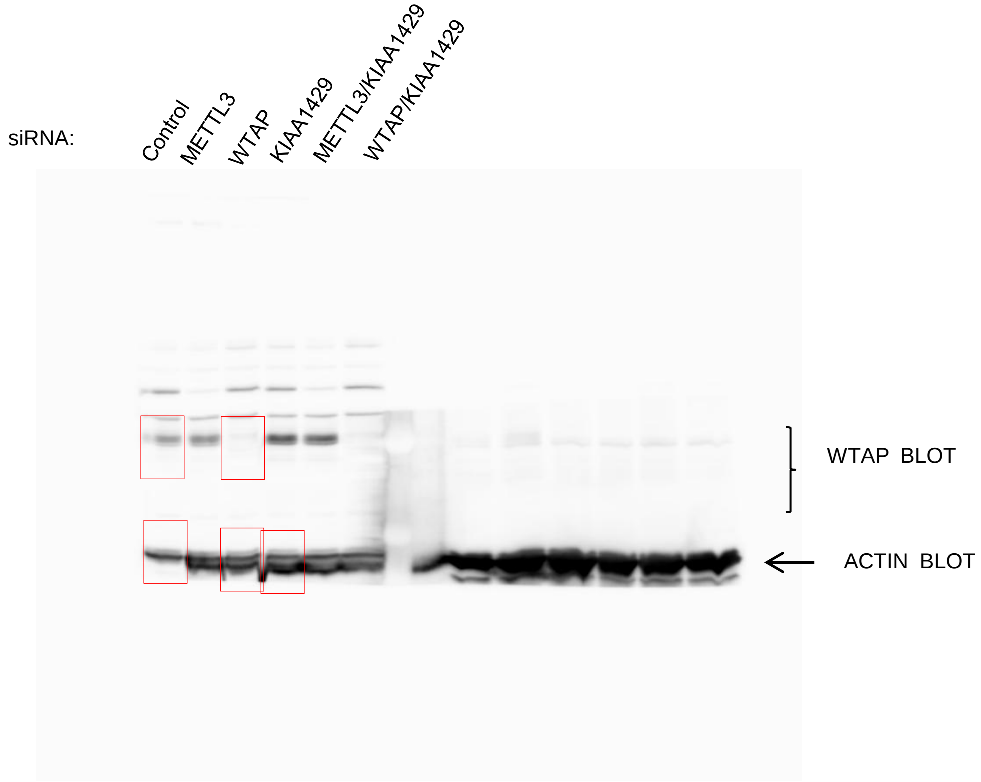

Original images of blots displayed in Fig 1C -lower panel.

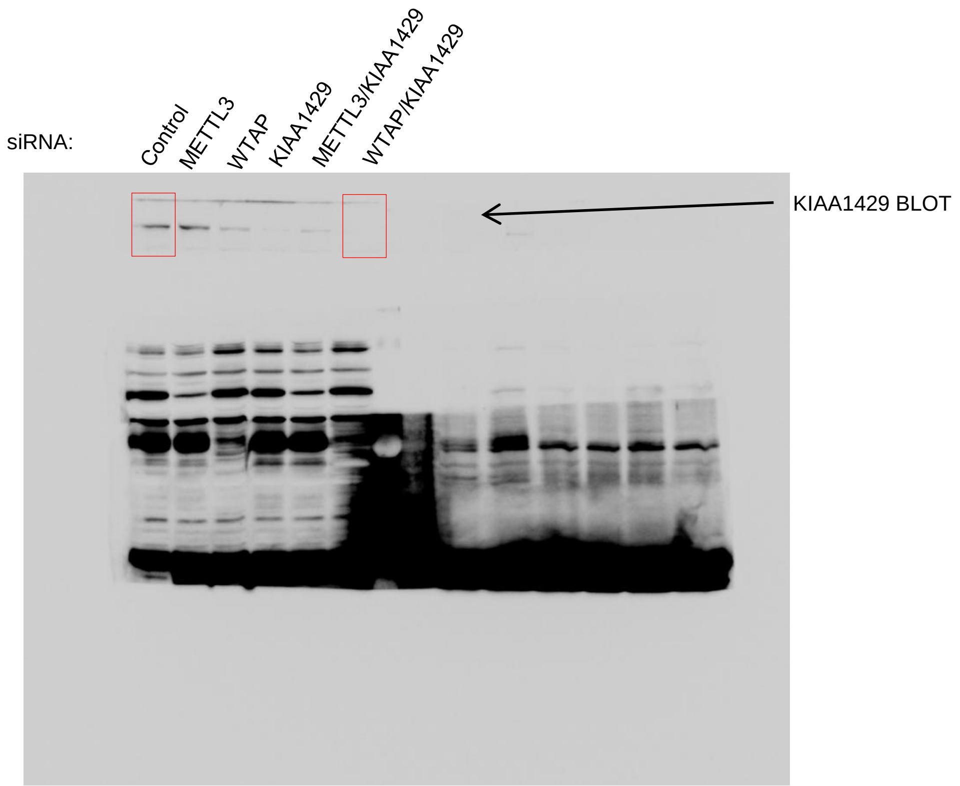

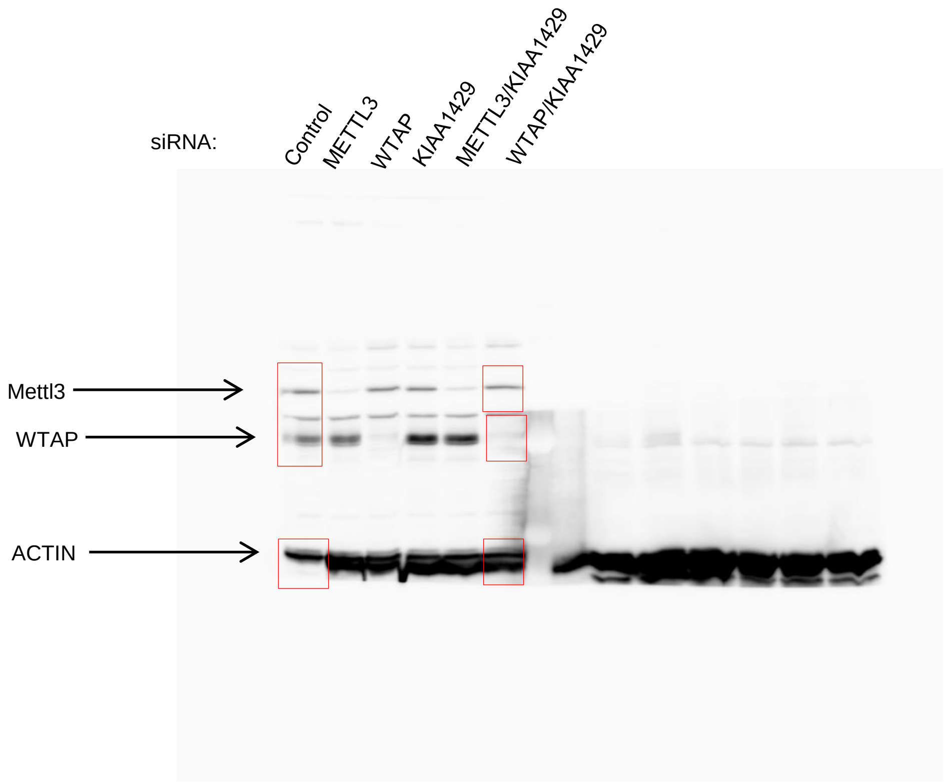

Original images of blots displayed in Fig 1D.

THOC5

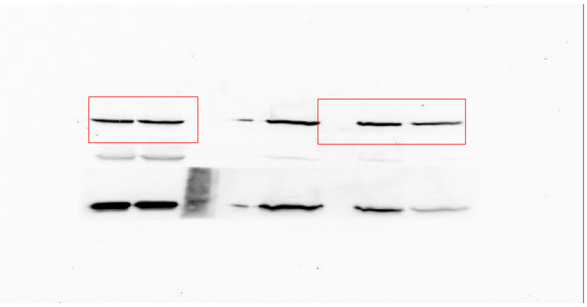

METTL3

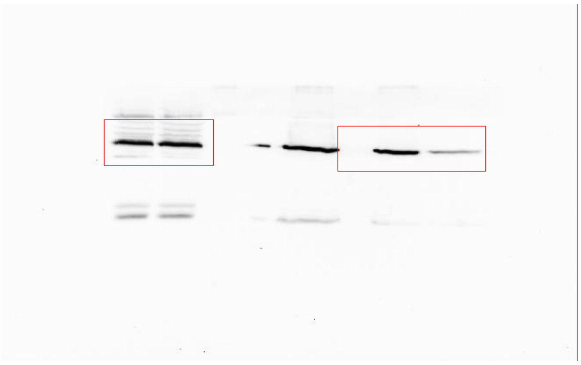

DDX39A/B

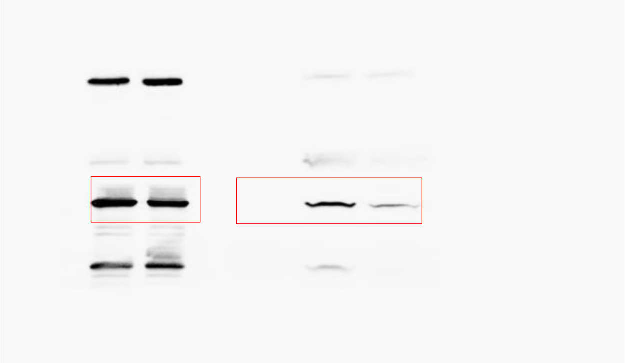

ALYREF

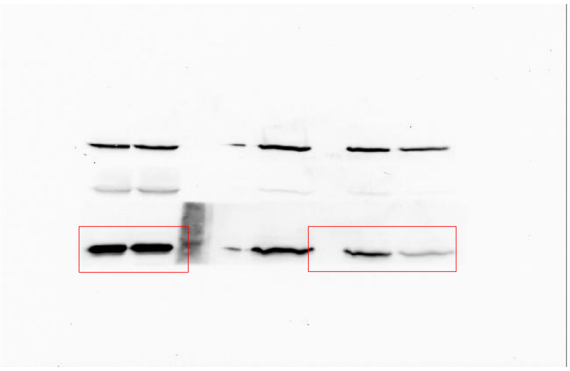

KIAA1429

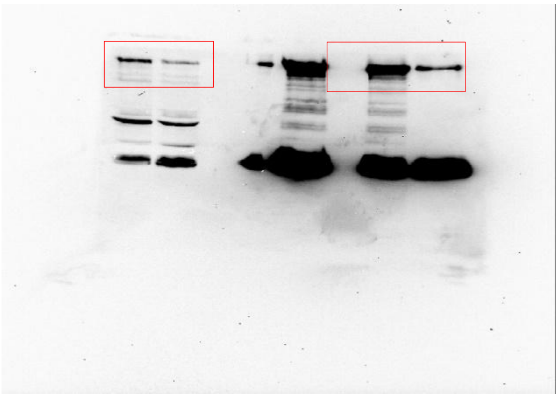

WTAP

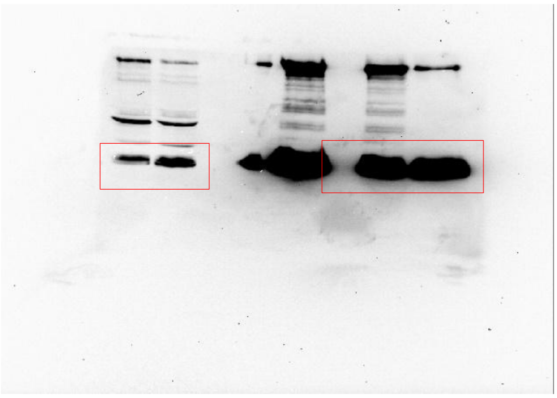

HNRNPA1

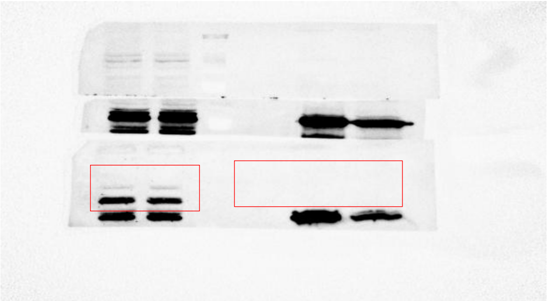

Original images of blots displayed in Fig 1E.

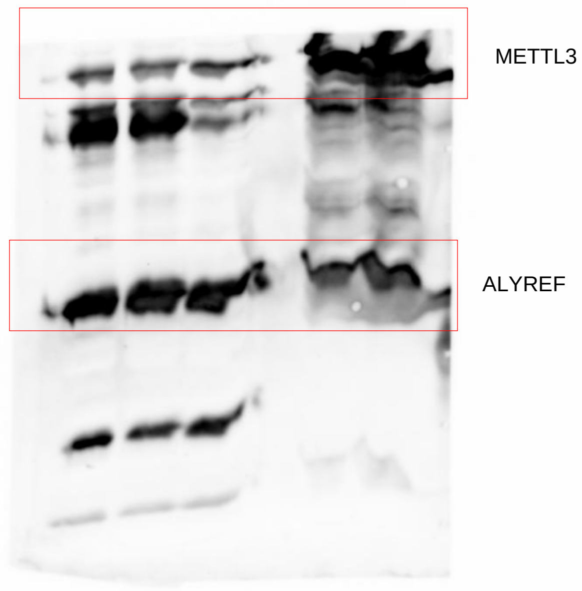

Original images of blots displayed in Fig 5B.

YTHDC1

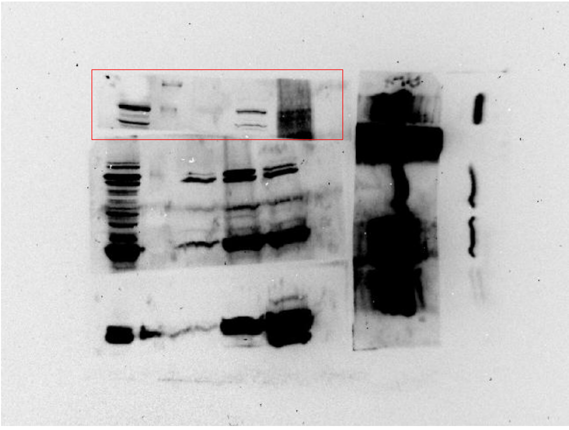

CHTOP

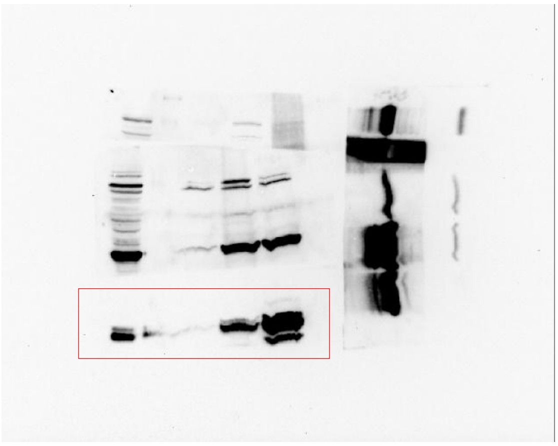

DDX39A/B

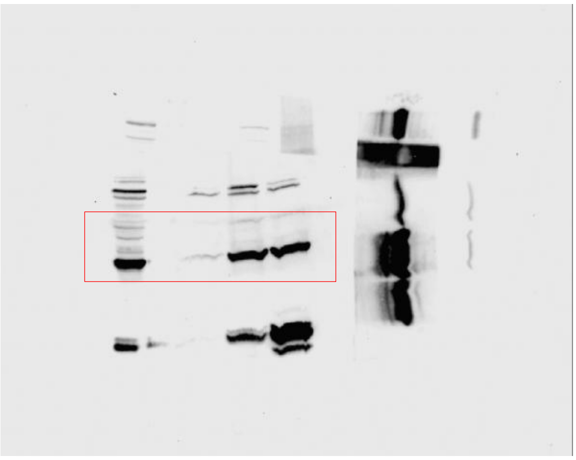

ALYREF

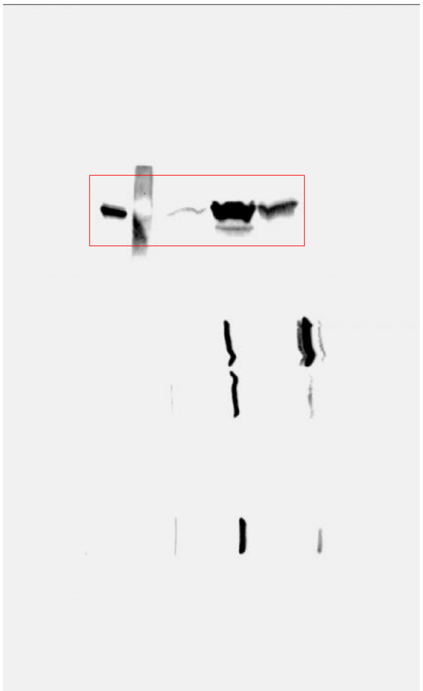

HNRNPA1

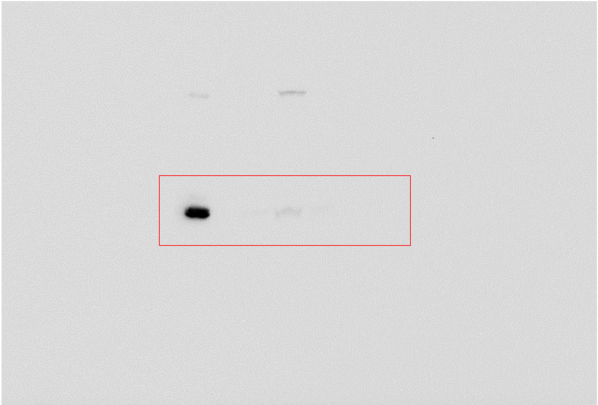

Original images of blots displayed in Fig 5C.

YTHDC1

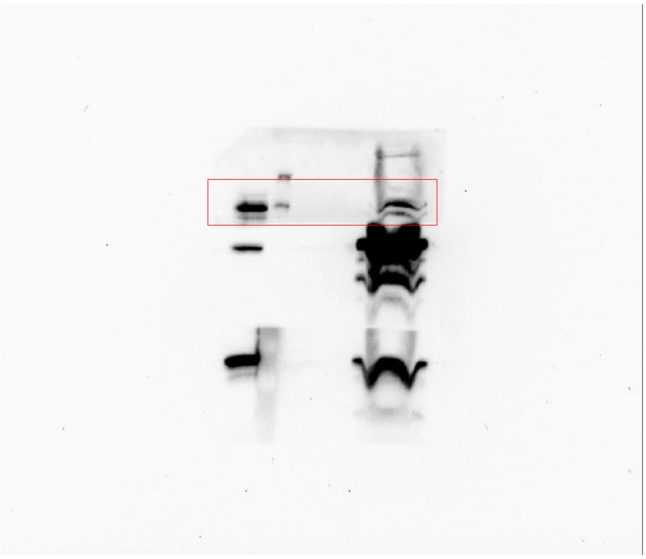

DDX39A/B

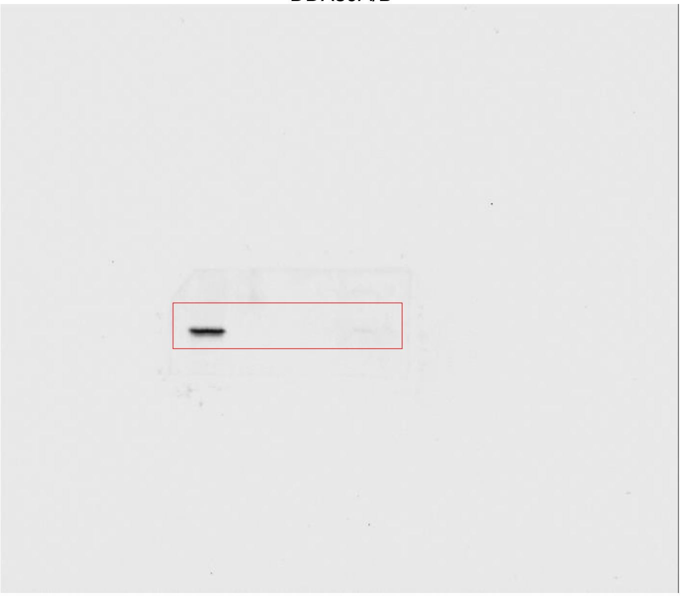

NXF1

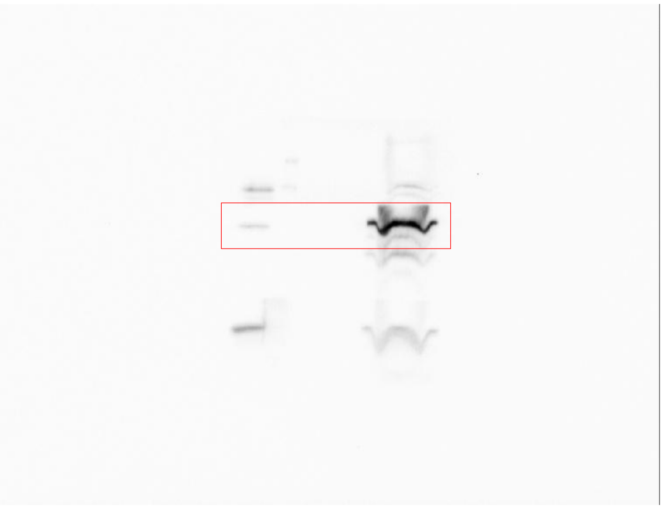

ALYREF

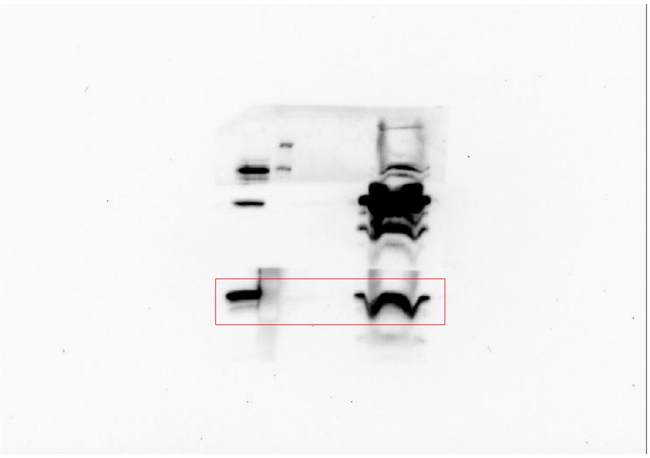

Original images of blots displayed in Fig 5D.

YTHDC1

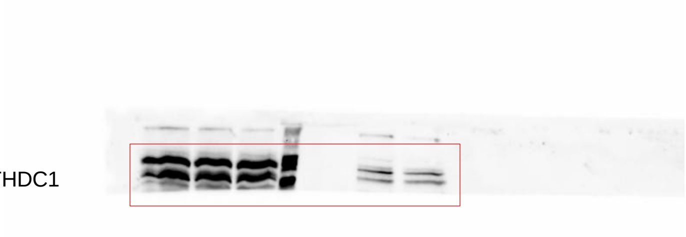

WTAP

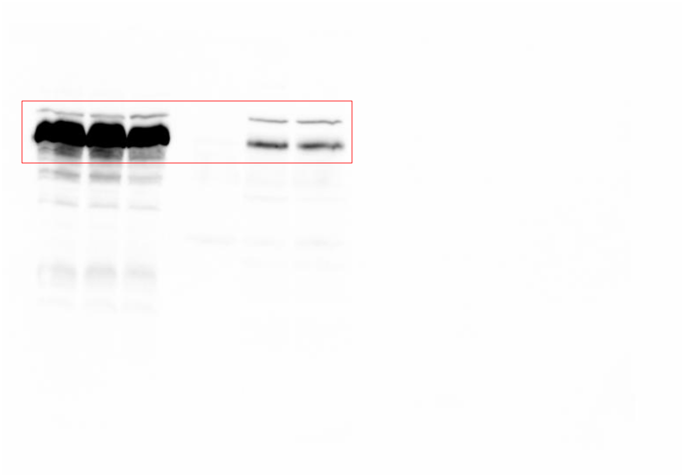

METTL3

METTL14

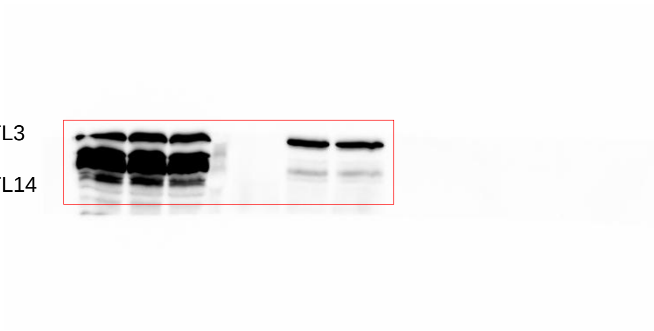

Original images of blots displayed in Fig 5E.

YTHDC1

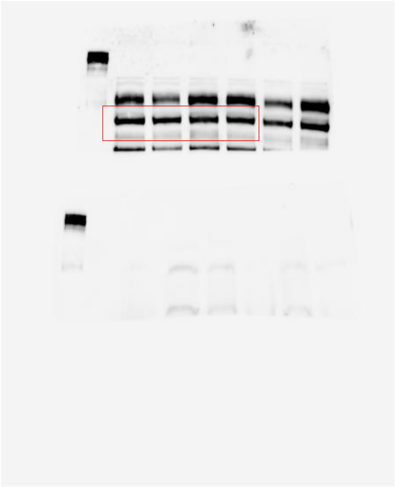

YTHDC1

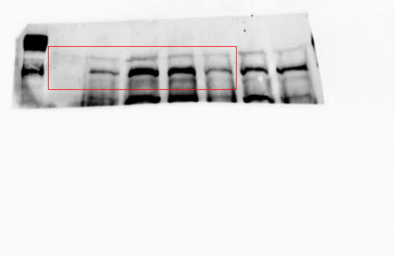

DDX39A/B

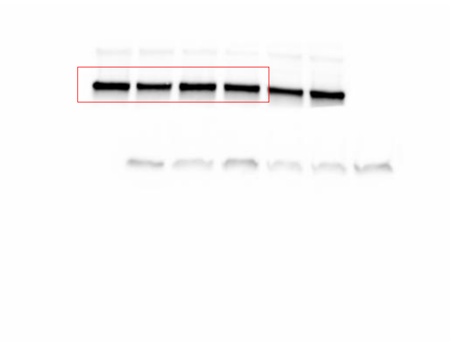

DDX39A/B

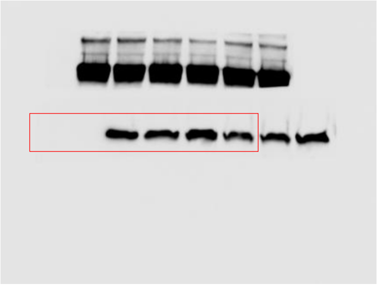

ALYREF

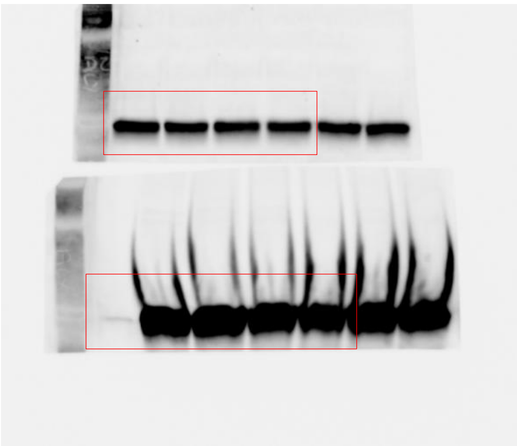

Original images of blots displayed in Fig 5F.

YTHDC1

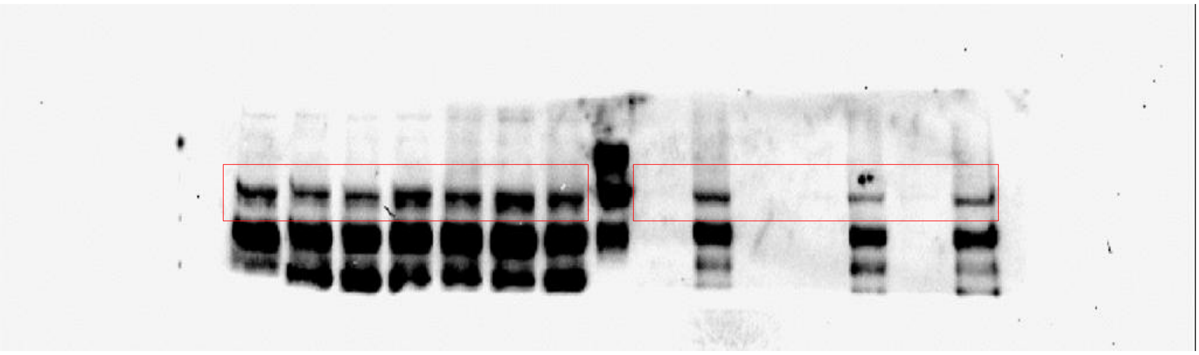

DDX39A/B

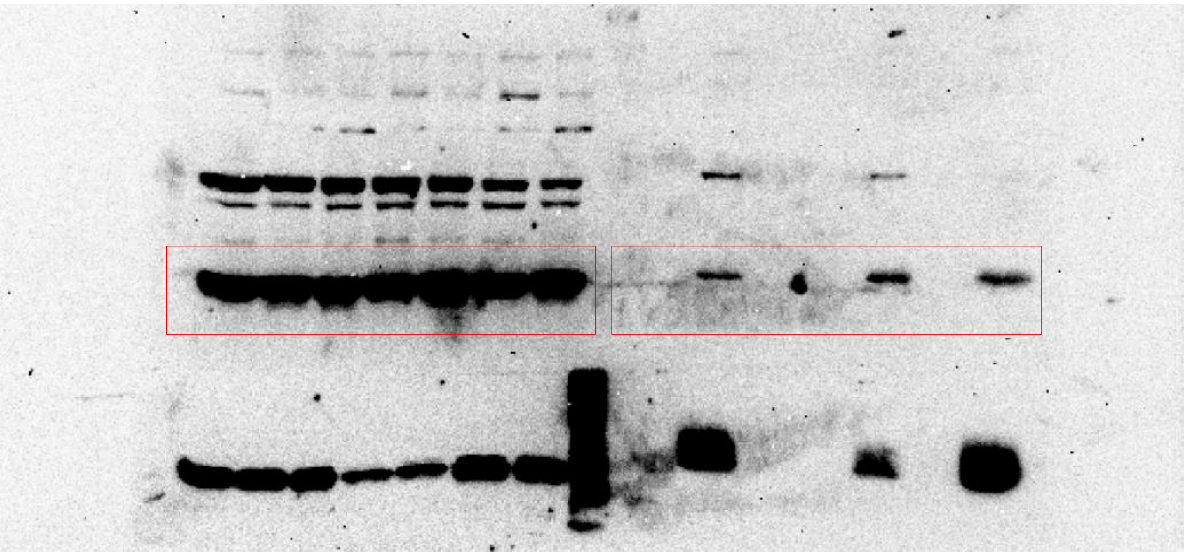

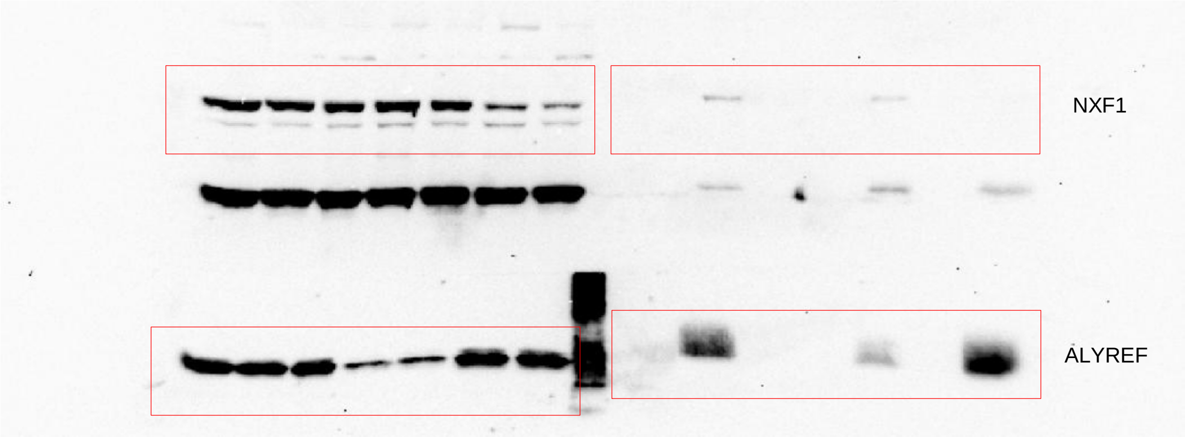

Original images of blots displayed in Fig S1A.

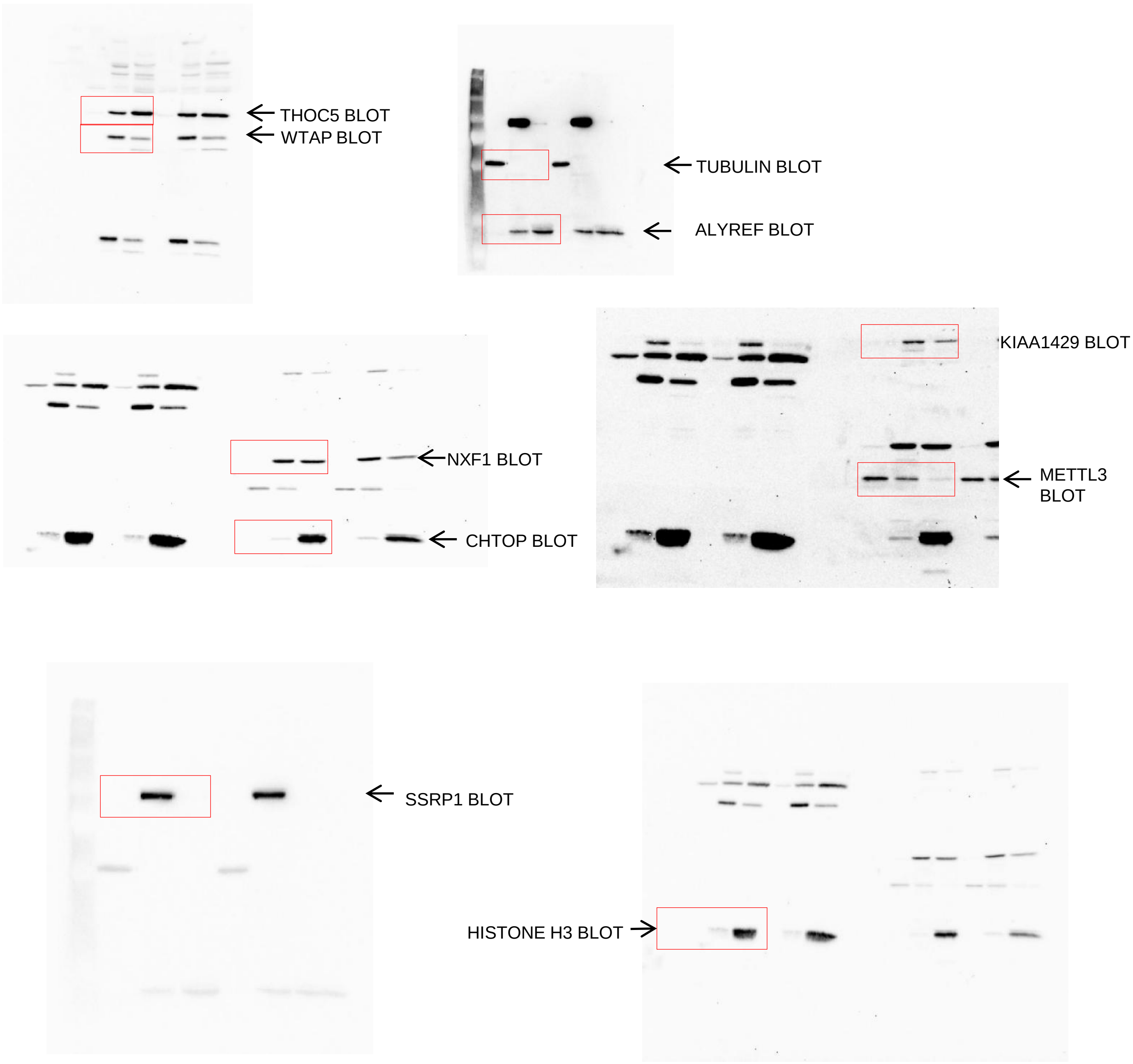

Original images of blots displayed in Fig S5A.

YTHDC1

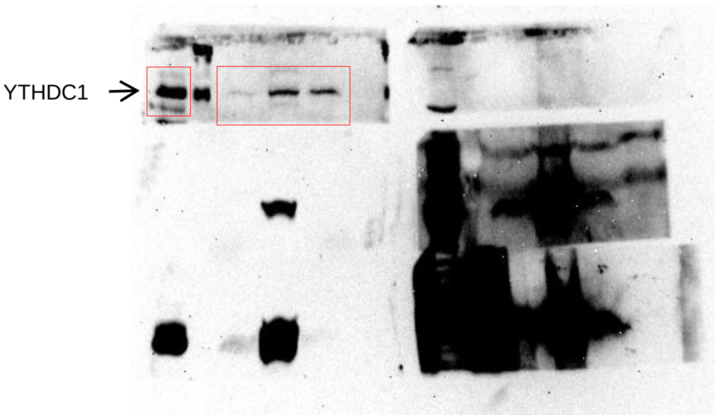

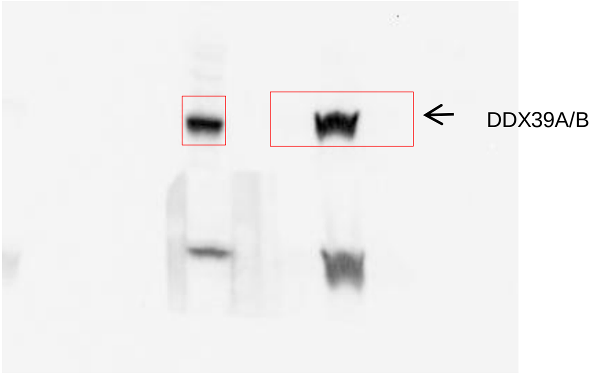

DDX39A/B

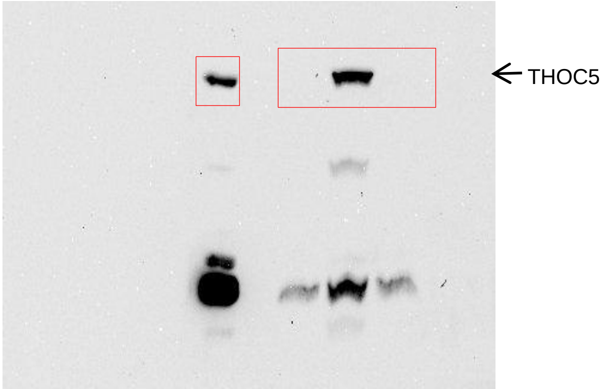

THOC5

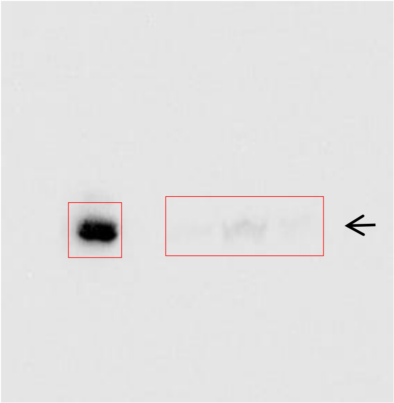

HNRNPA1

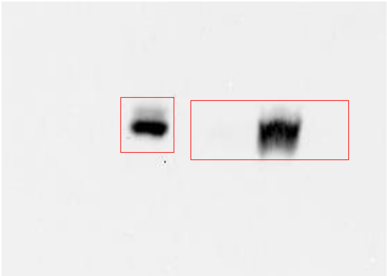

CHTOP

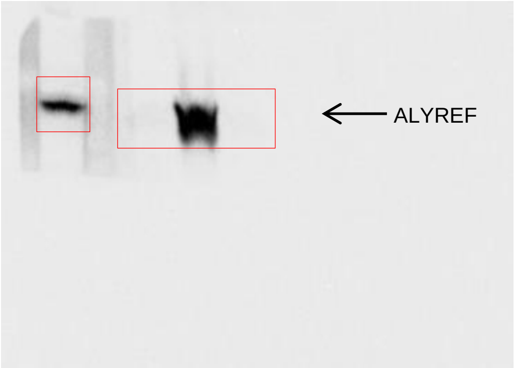

ALYREF

Original images of blots displayed in Fig S5B.

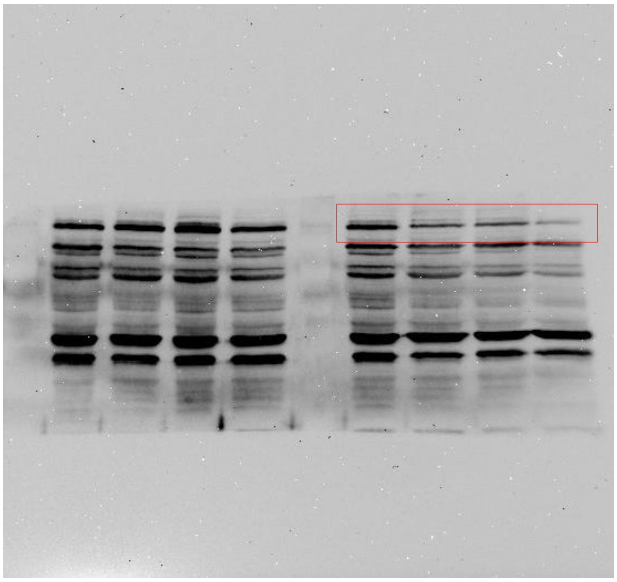

YTHDC1 BLOT

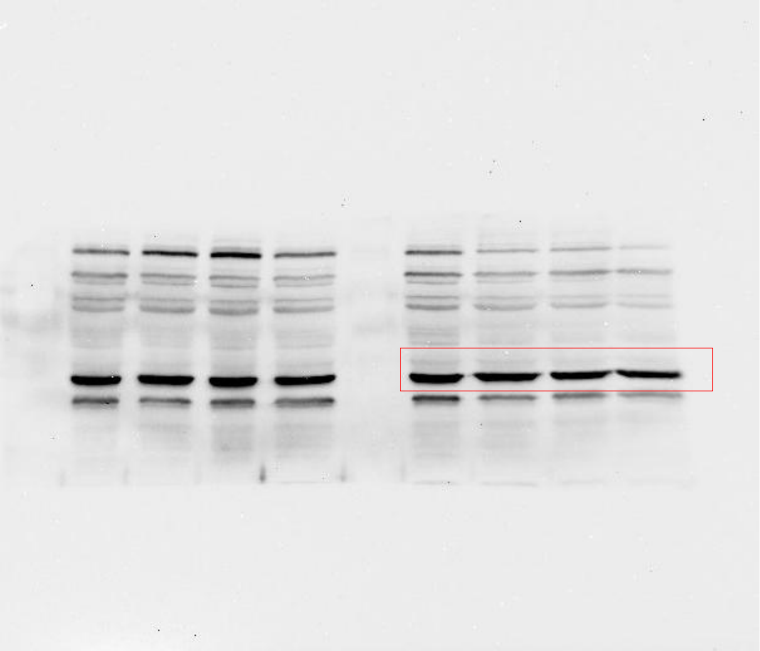

ACTIN BLOT
